# Supplementary material for: Regional heterogeneity in left atrial stiffness impacts passive deformation in a cohort of patient-specific models
Source: PLoS Comput Biol. 2025 Nov 5;21(11):e1013656. doi: 10.1371/journal.pcbi.1013656 (PMC12599961; doi:10.1371/journal.pcbi.1013656)
Supplement: S1 File — Description showing how LA blood pool, myocardium and EAT were segmented from patient images. (PDF) [file pcbi.1013656.s001.pdf]

Model generation from CT images

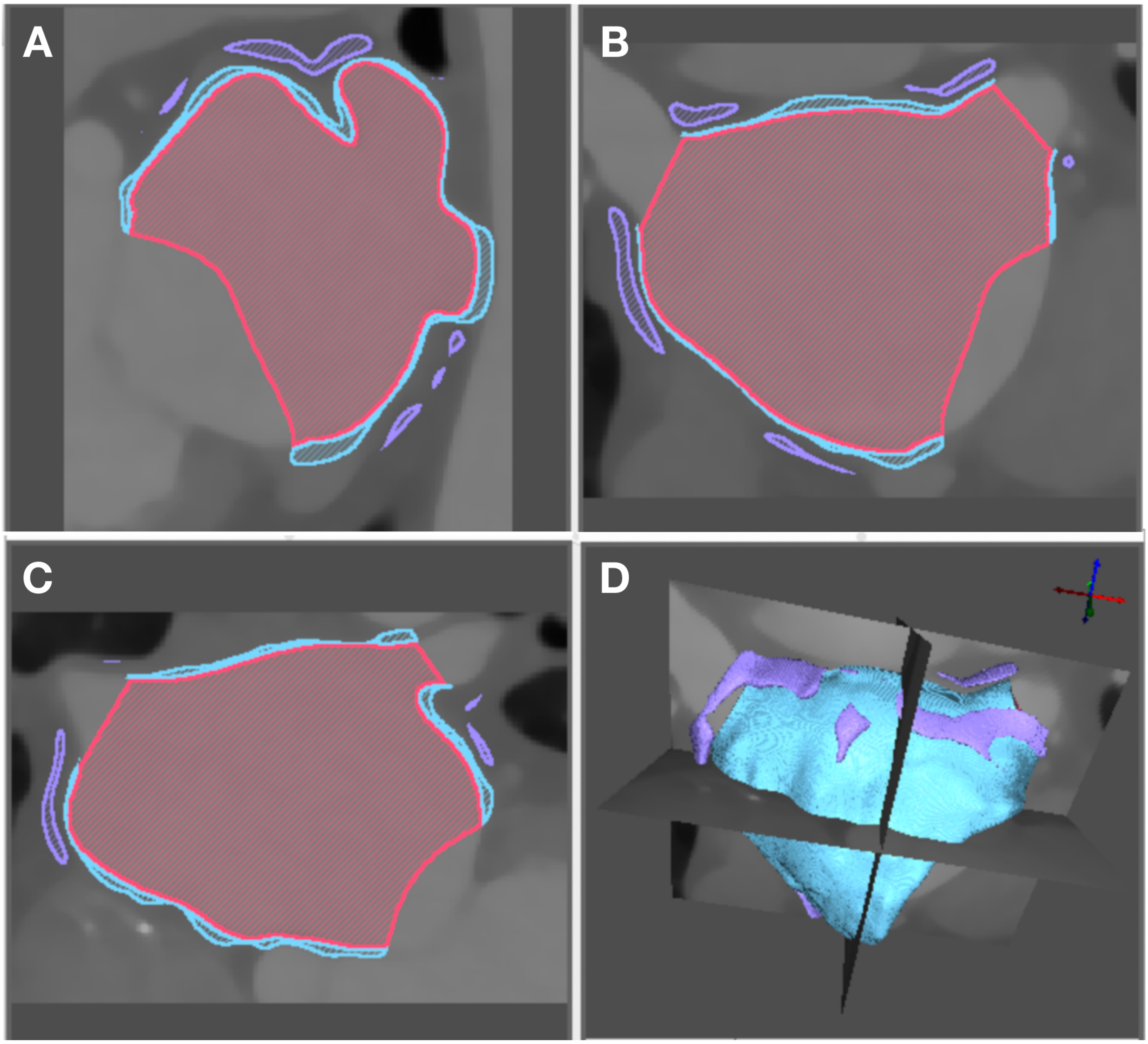

Fig 1: **LA anatomy from CT image.**2D and 3D views showing the LA bloodpool (pink), myocardium (blue) and EAT (purple) derived from the end-diastolic frame of gated CT image-set for a representative patient.

.
