# Supplementary material for: Regional heterogeneity in left atrial stiffness impacts passive deformation in a cohort of patient-specific models
Source: PLoS Comput Biol. 2025 Nov 5;21(11):e1013656. doi: 10.1371/journal.pcbi.1013656 (PMC12599961; doi:10.1371/journal.pcbi.1013656)
Supplement: S2 File — We compared the effects of EAT volume on atrial biomechanics using different methods of EAT quantification. (PDF) [file pcbi.1013656.s002.pdf]

Sensitivity of EAT quantification

Two HU ranges are commonly used to quantify EAT from cardiac CT images: −190 and −30 HU [1] and −195 and −45 HU [2]. Fig 1 shows the cohort of LA models used in this study with the surrounding EAT quantified using a HU range of −190 and −30 HU.

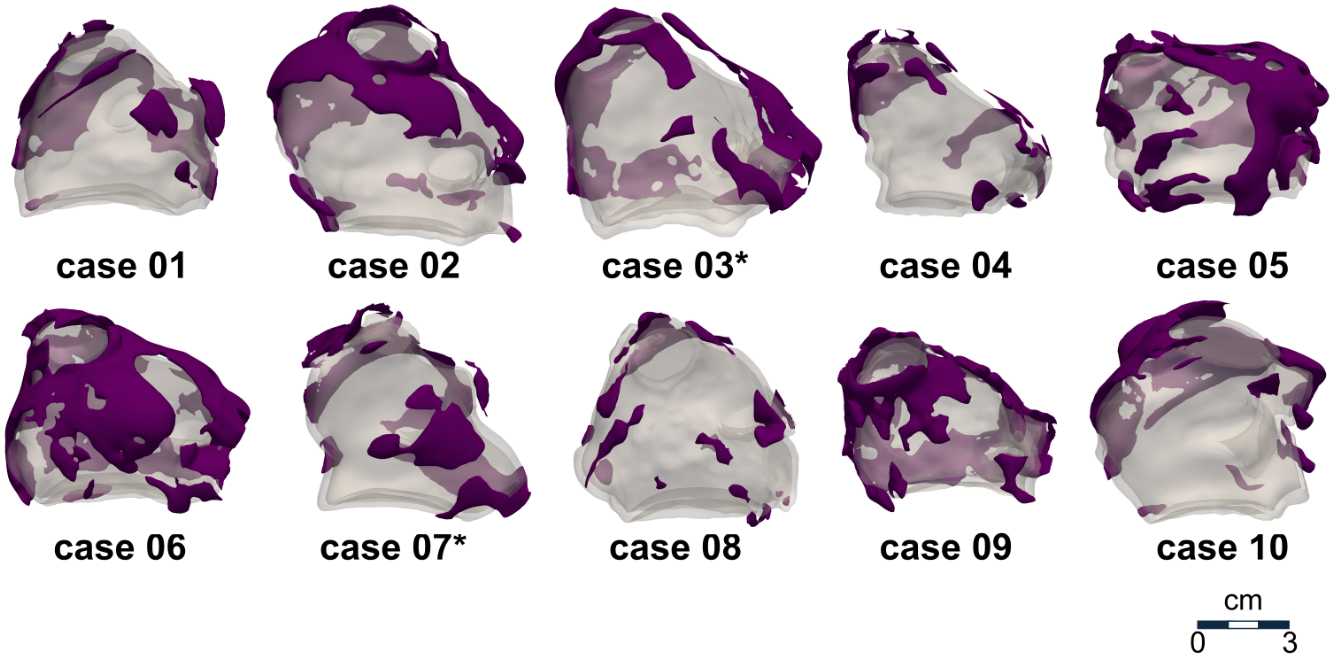

Fig 1: **Left atrial mesh cohort with surrounding EAT**. Images show the anterior view of the cohort of 10 meshes with EAT. * indicates patients with AF.

Here, we examine how the choice of HU range used to characterise EAT affects our findings. Over the cohort, there was a significant difference in the volume of EAT identified globally and in each of the five regions (Fig 2), with greater amounts of adipose tissue being identified when a wider HU range was used. Significance was determined in each region, using a paired t-test for dependent samples, with the Bonferroni correction for multiple comparisons applied.

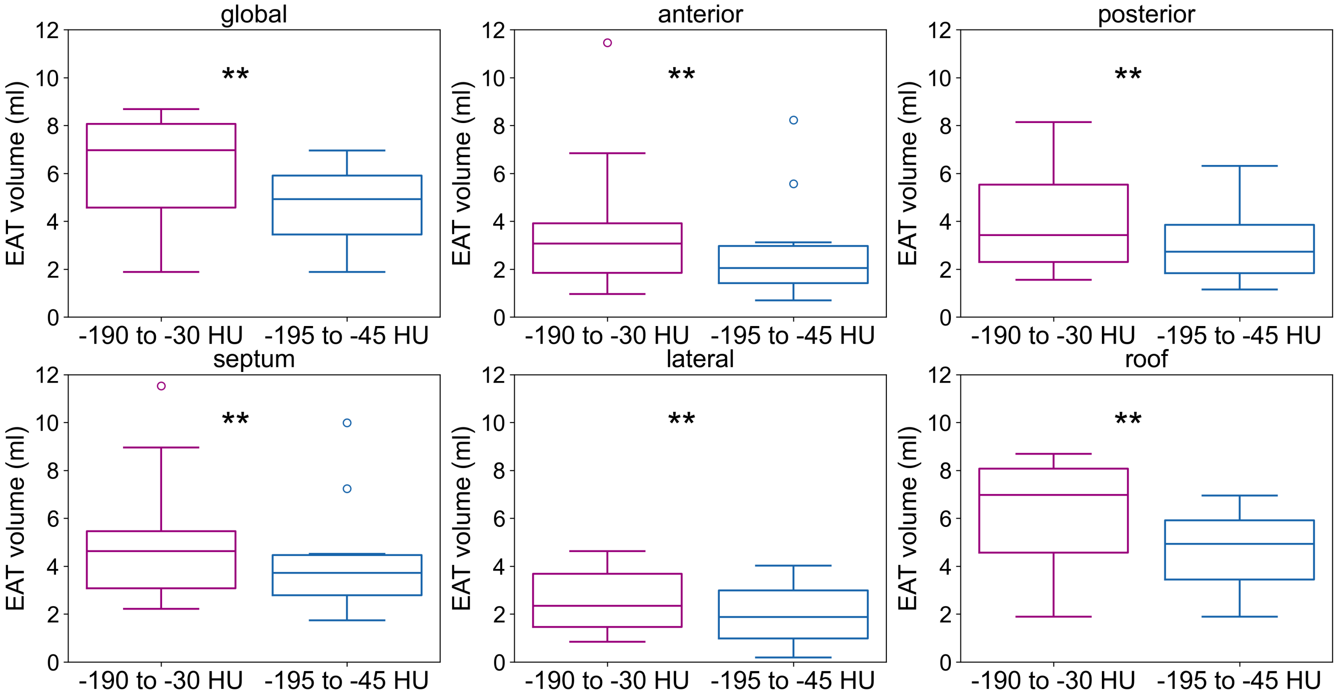

Fig 2: **Comparison of EAT volume per region**. Boxplots showing the difference in the EAT volume quantified when using two HU ranges commonly implemented in literature. ** indicates a p-value < 0.01.

Despite the difference in the volume of EAT identified between methods, the relationship between regional EAT and regional ES displacement or regional stiffness was maintained. We found that the regional EAT volume with a density of -195 and -45 HU was not a significant predictor of regional ES displacement ($p = 0.315$) or regional stiffness($p = 0.229$) in HF patients.

References

1. Monti CB, Codari M, De Cecco CN, Secchi F, Sardanelli F, Stillman AE. Novel imaging biomarkers: epicardial adipose tissue evaluation. The British Journal of Radiology. 2020;93(1113). doi:10.1259/BJR.20190770.

2. El Mahdiui M, Simon J, Smit JM, Kuneman JH, Van Rosendael AR, Steyerberg EW, et al. Posterior Left Atrial Adipose Tissue Attenuation Assessed by Computed Tomography and Recurrence of Atrial Fibrillation After Catheter Ablation. Circulation: Arrhythmia and Electrophysiology. 2021;14(4):E009135. doi:10.1161/CIRCEP.120.009135/SUPPL_FILE/CIRCAE_CIRCAE-2020-009135_SUPP1.PDF.
