# Supplementary material for: Regional heterogeneity in left atrial stiffness impacts passive deformation in a cohort of patient-specific models
Source: PLoS Comput Biol. 2025 Nov 5;21(11):e1013656. doi: 10.1371/journal.pcbi.1013656 (PMC12599961; doi:10.1371/journal.pcbi.1013656)
Supplement: S3 File — We compared model results using a high resolution, high computational resource mesh and a lower resolution, less computationally demanding mesh. (PDF) [file pcbi.1013656.s003.pdf]

Mesh convergence

To generate meshes from the labelled LA segmentation, we used the Computational Geometry Algorithm Library [1]. Meshing involved discretising the segmented LA geometry into tetrahedral mesh elements.

Initially, the atrial anatomies were meshed using tetrahedral elements with maximum edge length of 0.2 mm. This initial edge length was chosen to capture the thin-walled atrial anatomy. The mesh was then resampled using meshtool [2], an open-source software for mesh processing, so that the tetrahedral elements had an average edge length of 0.6 mm.

The edge length chosen for our simulations should concurrently satisfy both accuracy and efficiency of the simulation results. Therefore, we performed an analysis on the mesh convergence of the finite element model using the higher resolution mesh of maximum edge length 0.2 mm and the lower resolution of average edge length 0.6 mm.

In our mesh convergence analysis, we quantified the impact of mesh element refinement on the passive inflation of the ED mesh. The simulation set-up for the passive inflation and the simulation loading conditions are shown in Fig 1

Fig 2 show the volume, global and regional displacement traces derived from a passive inflation simulation carried out using both meshes. The difference between the simulated volumes and displacements at the two mesh resolutions are of the same order of magnitude of the expected uncertainty of the simulation results. Computation time increased from 23.3 minutes to 644.8 minutes when the higher resolution mesh was used - a 27.7- fold increase in compute time. Comparing the simulated outputs obtained using both meshes, the average difference in the global and regional displacements at ES was ± 0.22 mm while the discrepancy between the simulated volume at ES was ± 1 ml.

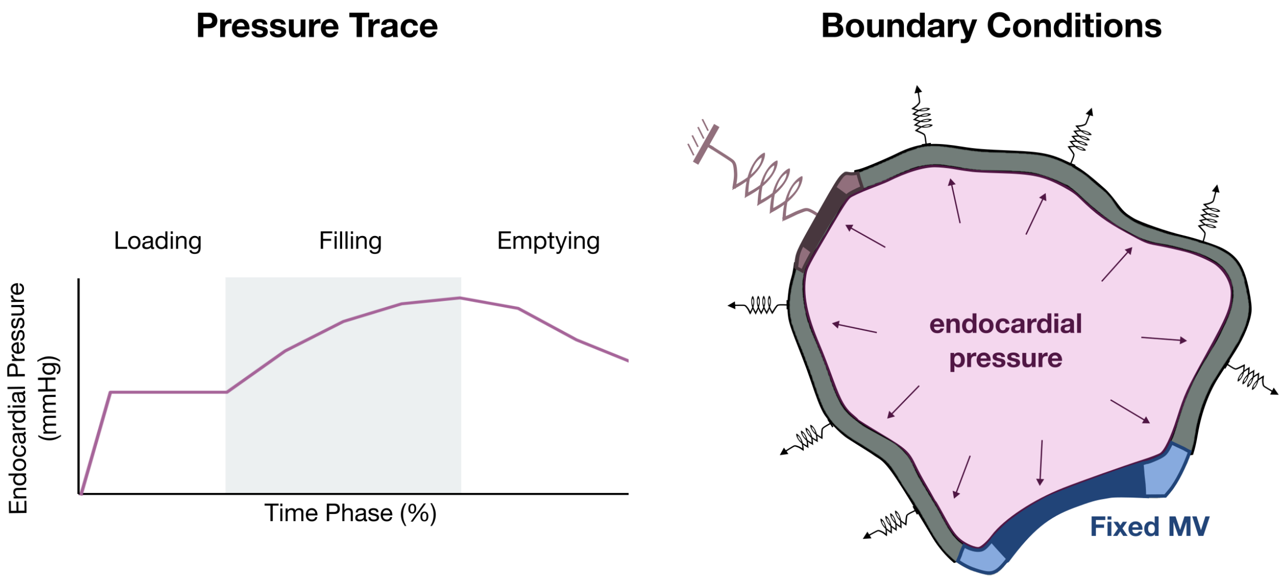

Fig 1: **Simulation set-up for mesh convergence study**. The LA MV annulus was fixed and the LA was passively inflated to from 4 mmHg to 34 mmHg.

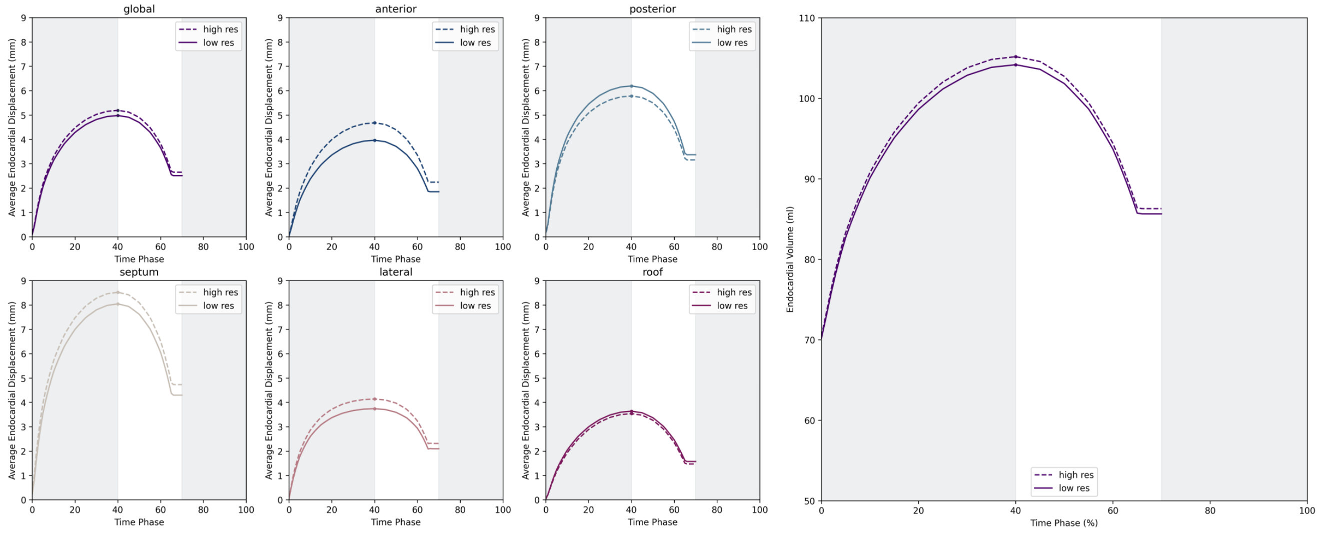

Fig 2: **Simulated LA features**. Global and regional displacement traces and volume traces obtained during a passive inflation simulation using a high resolution mesh with maximum edge length 0.2 mm and a lower resolution mesh with average edge length 0.6 mm.

References

1. The CGAL Project. CGAL User and Reference Manual. 5th ed. CGAL Editorial Board; 2023. Available from: `https://doc.cgal.org/5.6/Manual/packages.html`.
2. Neic A, Gsell MAF, Karabelas E, Prassl AJ, Plank G. Automating image-based mesh generation and manipulation tasks in cardiac modeling workflows using Meshtool. SoftwareX. 2020;11:100454. doi:10.1016/J.SOFTX.2020.100454.
