# Supplementary material for: Regional heterogeneity in left atrial stiffness impacts passive deformation in a cohort of patient-specific models
Source: PLoS Comput Biol. 2025 Nov 5;21(11):e1013656. doi: 10.1371/journal.pcbi.1013656 (PMC12599961; doi:10.1371/journal.pcbi.1013656)
Supplement: S4 File — Detailed explanation of how the five regions on the LA were defined using UACs. (PDF) [file pcbi.1013656.s004.pdf]

Region definition

The universal atrial coordinate (UAC) system [1] was used to divide the LA into five regions - anterior, posterior, septum, lateral and roof. The vein centres of the right superior and left inferior pulmonary veins in the endocardial UAC system were identified using the `pyvista` Python library. The UAC surface mesh was then split by drawing two perpendicular lines through the right superior and left inferior vein centres (Fig 1, panel A). Each surface mesh element was retagged to correspond to one of the five regions (Fig 1, panel B). These region element tags were then mapped on to the three-dimensional endocardial surface mesh Fig 1, panel C). Any mislabelled floating elements were corrected using an automated pipeline. Floating elements were located and the region label reassigned to match the region surrounding them. This ensured smooth and continuous region definitions on the three-dimensional surface mesh. The regions defined on the endocardial surface were then projected on to the volumetric LA mesh using a transmural Laplace-based coordinate system, where a value of 0 was assigned to the endocardium and 1 assigned to the epicardium [2].

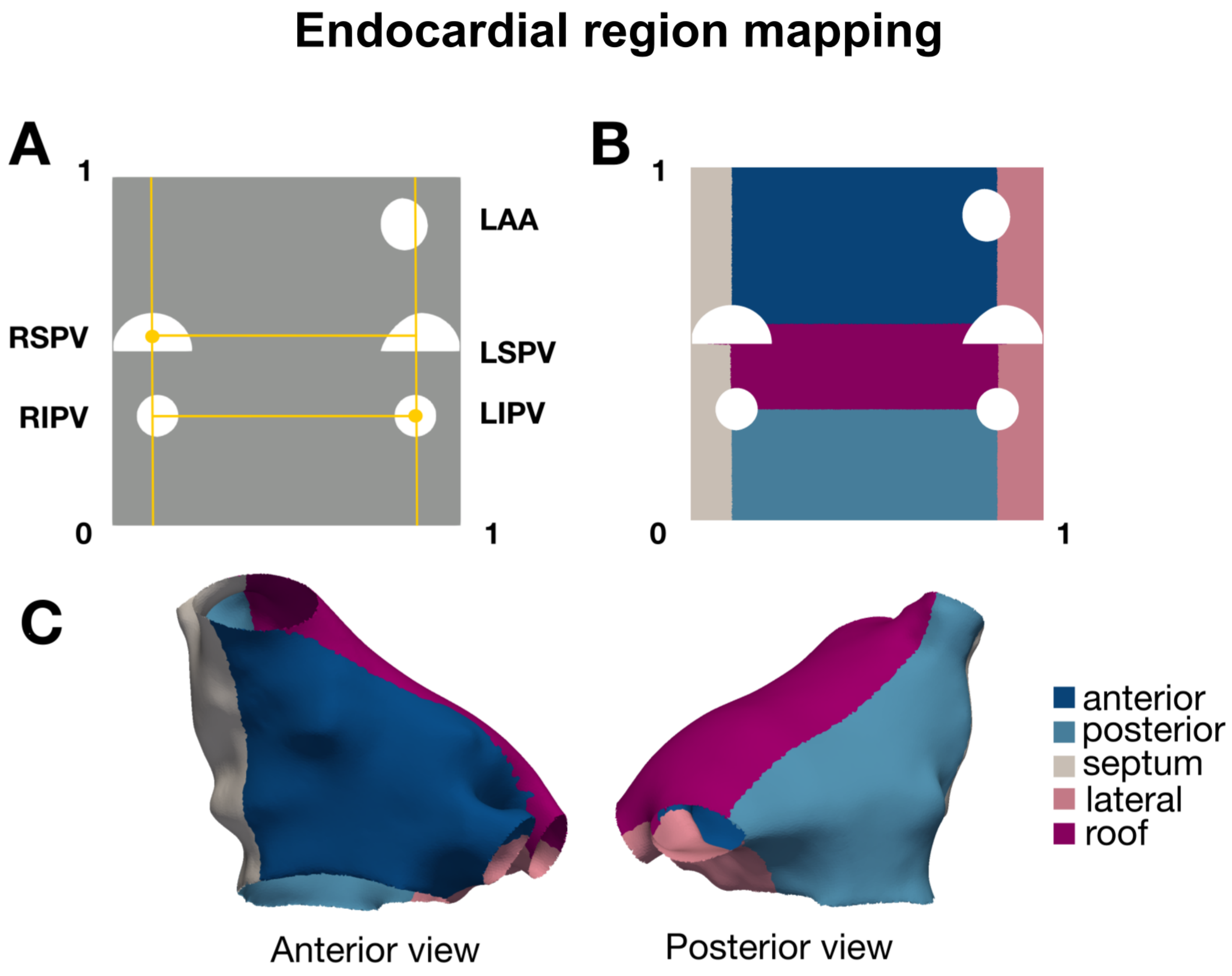

Fig 1: **LA region definition**. **A** Universal atrial coordinates (UACs) were used to separate the LA into 5 regions. The regions were created by drawing perpendicular lines through the center of right superior pulmonary vein (RSPV) and left inferior pulmonary vein (LIPV). **B** The 5 regions defined on the UAC representation of the LA endocardium. **C** The regions defined on the 3D LA endocardial mesh.

References

1. Roney CH, Pashaei A, Meo M, Dubois R, Boyle PM, Trayanova NA, et al. Universal atrial coordinates applied to visualisation, registration and construction of patient specific meshes. Medical Image Analysis. 2019;55:65–75. doi:10.1016/J.MEDIA.2019.04.004.

2. Bayer J, Prassl AJ, Pashaei A, Gomez JF, Frontera A, Neic A, et al. Universal ventricular coordinates: A generic framework for describing position within the heart and transferring data. Medical Image Analysis. 2018;45:83–93. doi:10.1016/J.MEDIA.2018.01.005.
