# Supplementary material for: Regional heterogeneity in left atrial stiffness impacts passive deformation in a cohort of patient-specific models
Source: PLoS Comput Biol. 2025 Nov 5;21(11):e1013656. doi: 10.1371/journal.pcbi.1013656 (PMC12599961; doi:10.1371/journal.pcbi.1013656)
Supplement: S5 File — We used the eikonal equation to obtain patient-specific LA wall thickness maps. We compared the performance of this fast-evaluating method to a slower, more computationally expensive but previously validated approach. (PDF) [file pcbi.1013656.s005.pdf]

Wall thickness calculation

In this study, the eikonal equation was used to estimate the thickness of the left atrial wall across the entire chamber. Using the cardiac arrhythmia research package (CARP), all the vertices on the epicardial surface were activated simultaneously, with the wavefront having a constant, isotropic conduction velocity of 1 mm/s. The thickness of the LA wall was then estimated as the wavefront arrival time at the endocardium. This method provided a fast and computationally cheap alternative to the method proposed by Bishop et al [1].

In Bishop et al., the Laplace equation was solved over a finite-element LA mesh. Local wall thickness measurements were then estimated from the length of field lines derived the Laplace equation and spanning the endocardium to epicardium domain. This method was validated against an LA phantom and accurately calculated wall thicknesses between 0.5 to 5.0 mm within errors less than 0.2 mm [1].

Comparing wall thickness measurements calculated using the eikonal-based and Laplace-based methods, we saw a mean error of 0.13 mm between the regional averages computed using both methods for 2 patient cases. Fig 1 shows a Bland-Altman plot and histogram comparing the two wall thickness calculation approaches. These results suggest good agreement between the methods.

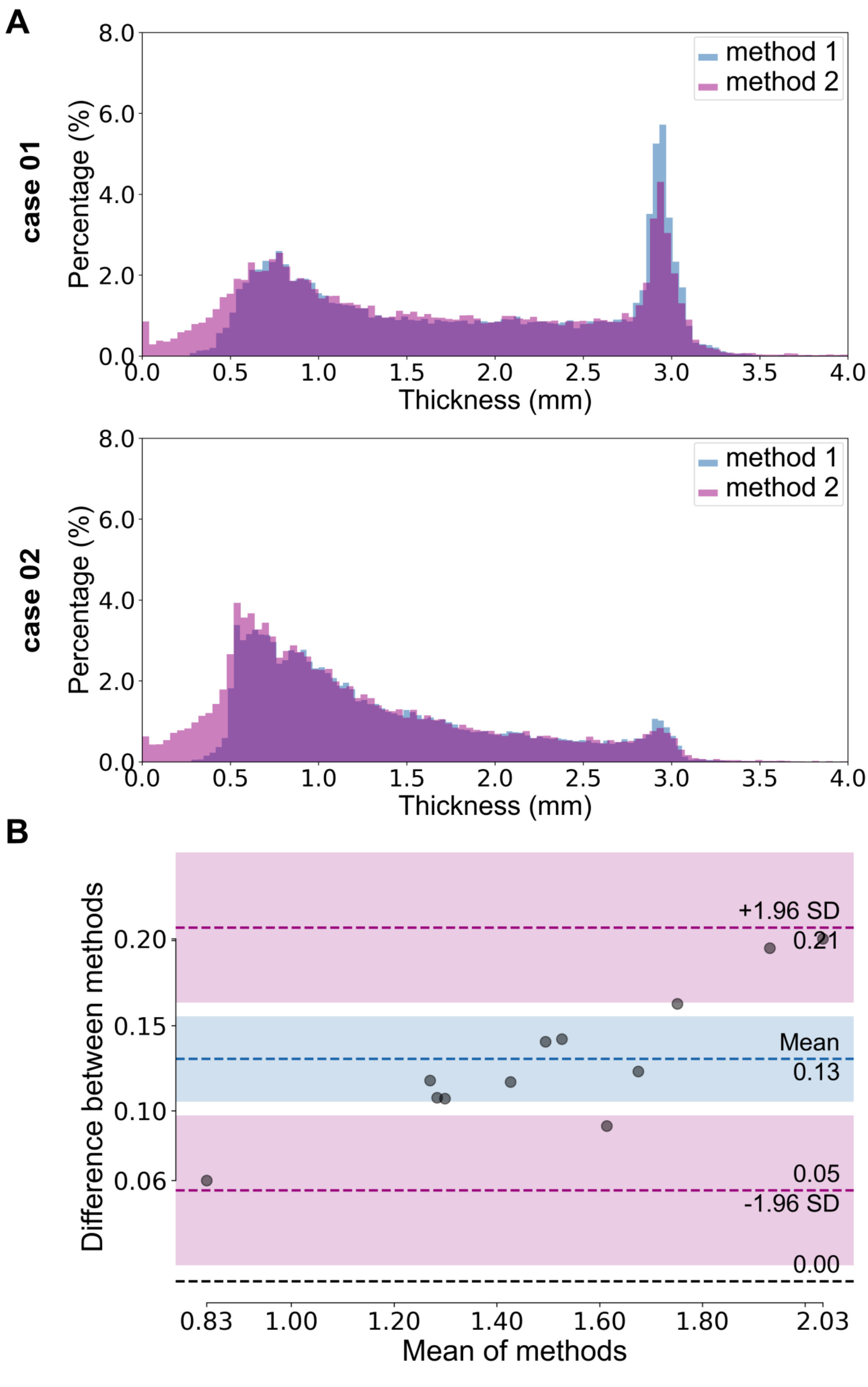

Fig 1: **Thickness metric comparison** The histograms show the distribution of thickness values calculated at each element using the fast eikonal-based method (method 1) and the computationally expensive Laplace method (method 2) for 2 representative cases. The Bland Altman plot shows that the mean difference between the regionally averaged wall thickness calculations between methods was 0.13 mm using 2 representative cases.

References

1. Bishop M, Rajani R, Plank G, Gaddum N, Carr-White G, Wright M, et al. Three-dimensional atrial wall thickness maps to inform catheter ablation procedures for atrial fibrillation. EP Europace. 2016;18(3):376–383. doi:10.1093/EUROPACE/EUV073.
