# Supplementary material for: Regional heterogeneity in left atrial stiffness impacts passive deformation in a cohort of patient-specific models
Source: PLoS Comput Biol. 2025 Nov 5;21(11):e1013656. doi: 10.1371/journal.pcbi.1013656 (PMC12599961; doi:10.1371/journal.pcbi.1013656)
Supplement: S6 File — Detailed explanation of the pericardium definition. (PDF) [file pcbi.1013656.s006.pdf]

Pericardium penalty map

In our simulator, the effect of the pericardium was modelled using normal springs with spatially varying stiffness on the surface of the epicardium as described in Strocchi et al. [1].

In Strocchi et al, LA motion, derived from a gated CT image set, was used to define the spatial variation in pericardium spring stiffness. The LA was divided into 18 regions. The image-derived ES displacements of the LA epicardial surface were extracted, interpolated over the surface triangles and projected normal to the surface. The displacement normal to the epicardial surface was normalised between 0 and 1 across the chamber for each patient. The average and standard deviation of the mean normalised displacement of each LA region was computed throughout the cohort. Through this analysis, Strocchi found that on average, the roof of the LA moves the least while the regions surrounding the mitral valve move the most [2].

In modelling the pericardium, normal springs restricted outward displacement in the direction normal to the epicardial surface while enabling frictionless sliding of the epicardium against the pericardium. Based on the averaged image-derived displacement data, the scaling map for the pericardial spring stiffness was defined such that epicardial regions with low and high displacement normal to the surface were applied with maximum and minimum penalty, respectively. As such, the maximum constraint was on the LA roof and while no constraint was applied to the area of the LA around the mitral valve, corresponding to [2]. In Strocchi et al., a smooth pericardium penalty function was used to scale the spring stiffness, k_{peri} over the chamber. The function was originally derived from motion data for the ventricles, similar to that described above [3]. These springs are applied as a traction on the surface elements. The traction exerted by these springs was defined as:

$$\mathbf{t}_N(\mathbf{u}(\mathbf{X},t),\mathbf{X}) = -k_{\text{peri}}(\mathbf{X})\left[(\mathbf{u}(\mathbf{X},t)-\mathbf{w}(\mathbf{X}))\cdot\frac{\mathbf{F}^{-T}\mathbf{N}(\mathbf{X})}{|\mathbf{F}^{-T}\mathbf{N}(\mathbf{X})|}\right]\frac{\mathbf{F}^{-T}\mathbf{N}(\mathbf{X})}{|\mathbf{F}^{-T}\mathbf{N}(\mathbf{X})|} \quad \text{on } \Gamma_{\text{PERI}}. \tag{1}$$

The function $k_{peri}(\mathbf{X})$ is used to represent local spatial effects of the pericardium. $\mathbf{u}(\mathbf{X},t)$ and $\mathbf{w}(\mathbf{X})$ represent the displacement of the surface nodes with respect to the stress-free configuration and to the reference configuration for the springs, respectively. $\mathbf{F}$ represents the deformation tensor and $\mathbf{N}(\mathbf{X})$ is the normal direction of boundary surface elements of the pericardial surface, Γ_{PERI}.

Fig 1 shows an example of the pericardium penalty of one of the cohort meshes. The pericardium penalty threshold (PTH) indicates the limit of the effect of the pericardium and was included as a parameter in the calibration procedure.

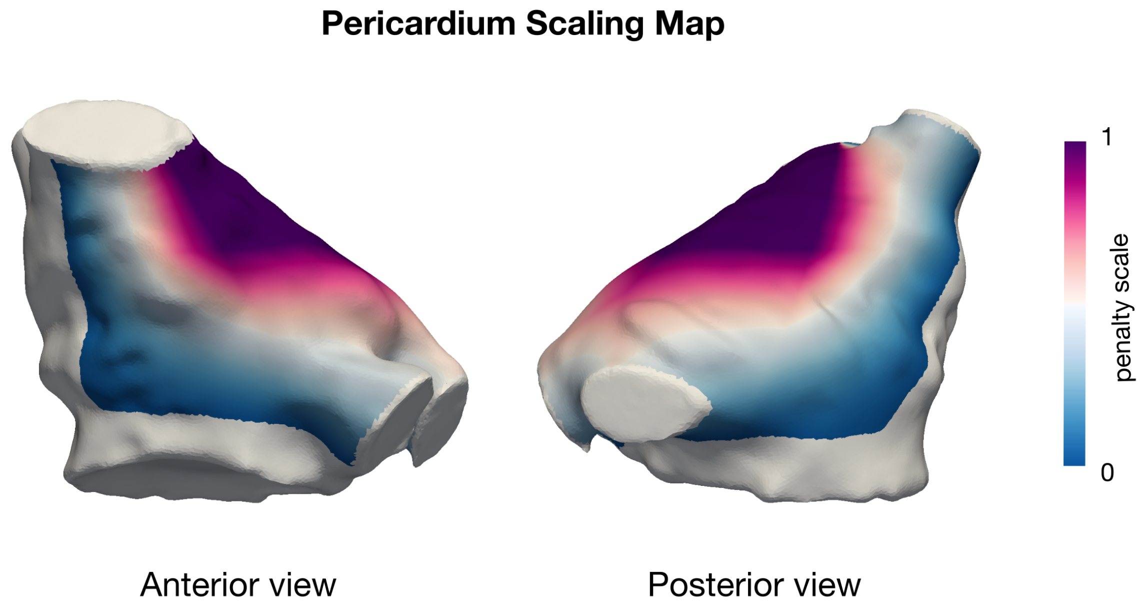

Fig 1: **Pericardium penalty map**. The penalty map scales the normal spring stiffness for the effect of the pericardium. The highest penalty is applied to the roof region while no penalty is applied in the region surrounding the MV annulus. The pericardium penalty threshold (PTH) is indicated by the limit of the coloured region on the mesh on the anterior and posterior regions and represents the boundary beyond which no effect of the pericardium is applied to the LA epicardium. No penalty is applied to the septal region.

References

1. Strocchi M, Gsell MAF, Augustin CM, Razeghi O, Roney CH, Prassl AJ, et al. Simulating ventricular systolic motion in a four-chamber heart model with spatially varying robin boundary conditions to model the effect of the pericardium. Journal of Biomechanics. 2020;101:109645. doi:10.1016/J.JBIOMECH.2020.109645.

2. Strocchi M, Augustin CM, Gsell MAF, Karabelas E, Neic A, Gillette K, et al. The Effect of Ventricular Myofibre Orientation on Atrial Dynamics. Lecture Notes in Computer Science (including subseries Lecture Notes in Artificial Intelligence and Lecture Notes in Bioinformatics). 2021;12738 LNCS:659–670. doi:10.1007/978-3-030-78710-3_63/FIGURES/4.

3. Strocchi M, Augustin CM, Gsell MAF, Karabelas E, Neic A, Gillette K, et al. A publicly available virtual cohort of fourchamber heart meshes for cardiac electromechanics simulations. PLoS ONE. 2020;15(6). doi:10.1371/journal.pone.0235145.
