# Supplementary material for: Regional heterogeneity in left atrial stiffness impacts passive deformation in a cohort of patient-specific models
Source: PLoS Comput Biol. 2025 Nov 5;21(11):e1013656. doi: 10.1371/journal.pcbi.1013656 (PMC12599961; doi:10.1371/journal.pcbi.1013656)
Supplement: S7 File — We used GSA to identify the most relevant regional stiffness parameters in each case of the 10-patient cohort. (PDF) [file pcbi.1013656.s007.pdf]

Passive mechanics sensitivity analysis

We are unable to uncouple the C and α stiffness parameters in the reformulated Guccione law given in the main text. Thus, we used GSA to exclude the unimportant regional stiffness parameters prior to our fitting procedure.

For the GSA, we carried out a Sobol' variance-based sensitivity analysis [1] using the Saltelli method [2] implemented in the `SALib` Python library [3]. Each input parameter was ranked by their importance across all the output features considered. This was done by computing the maximum total effect for each input across all outputs and normalising so that the maximum total effects for each parameter summed to 1, as shown in [4]. Fig 1 provides a comparison of the relative importance of regional C and α parameters over all cases in our cohort. These findings show that the sensitivity of regional α parameters tend to outrank regional C parameters across our cohort of heart failure patients. Based on this, for each case, we chose the fix C in all regions and fitted only regional α parameters.

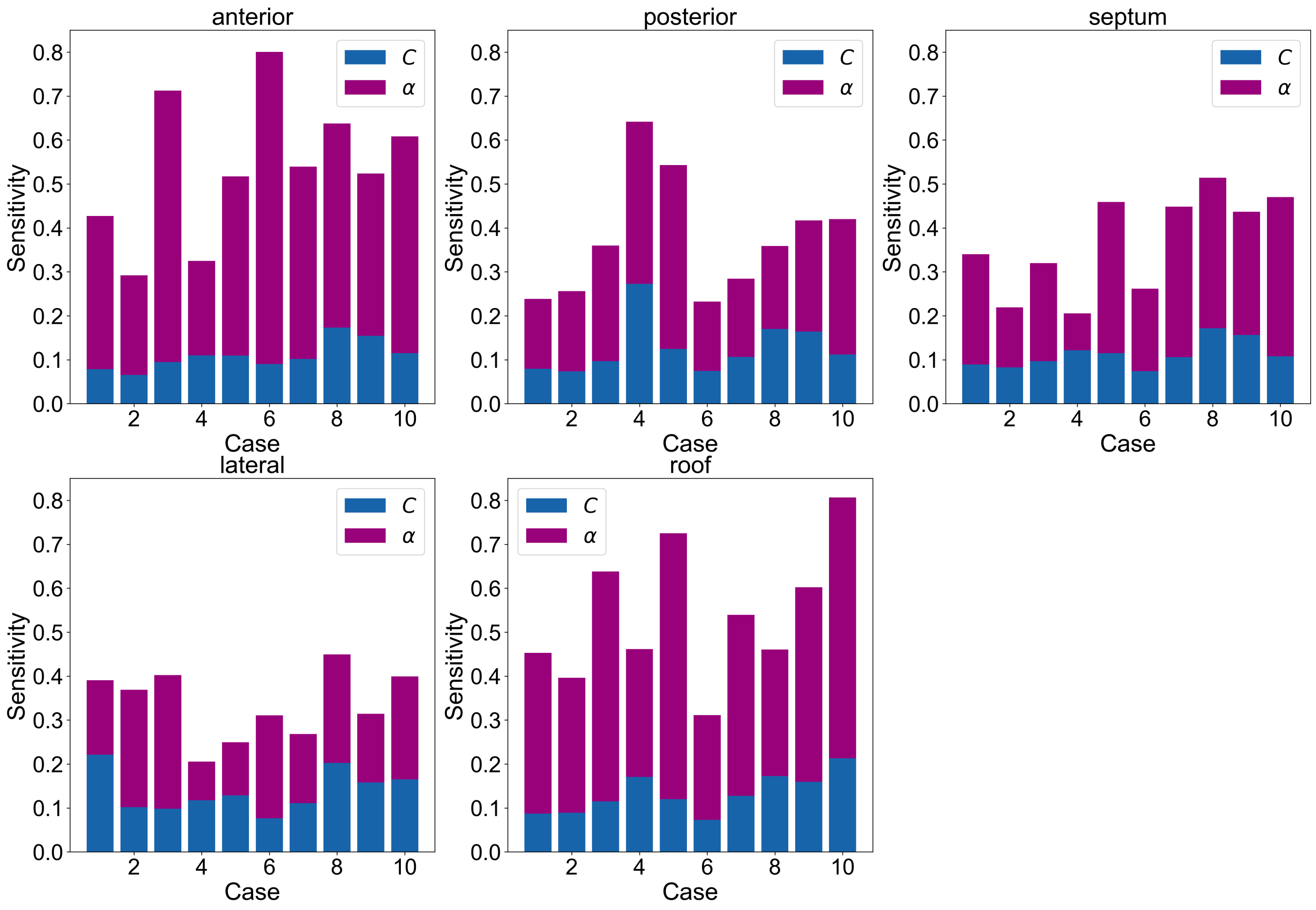

Fig 1: **Relative sensitivity of regional stiffness parameters**. Stacked barplots show the relative importance of the maximum sensitivity of C to α parameters determined from GSA in each region (anterior, posterior, septum, lateral, roof) across our 10-patient cohort.

The following figures (Fig 2 - 11) show the results of the GSA for each of the 10 patient cases in our cohort.

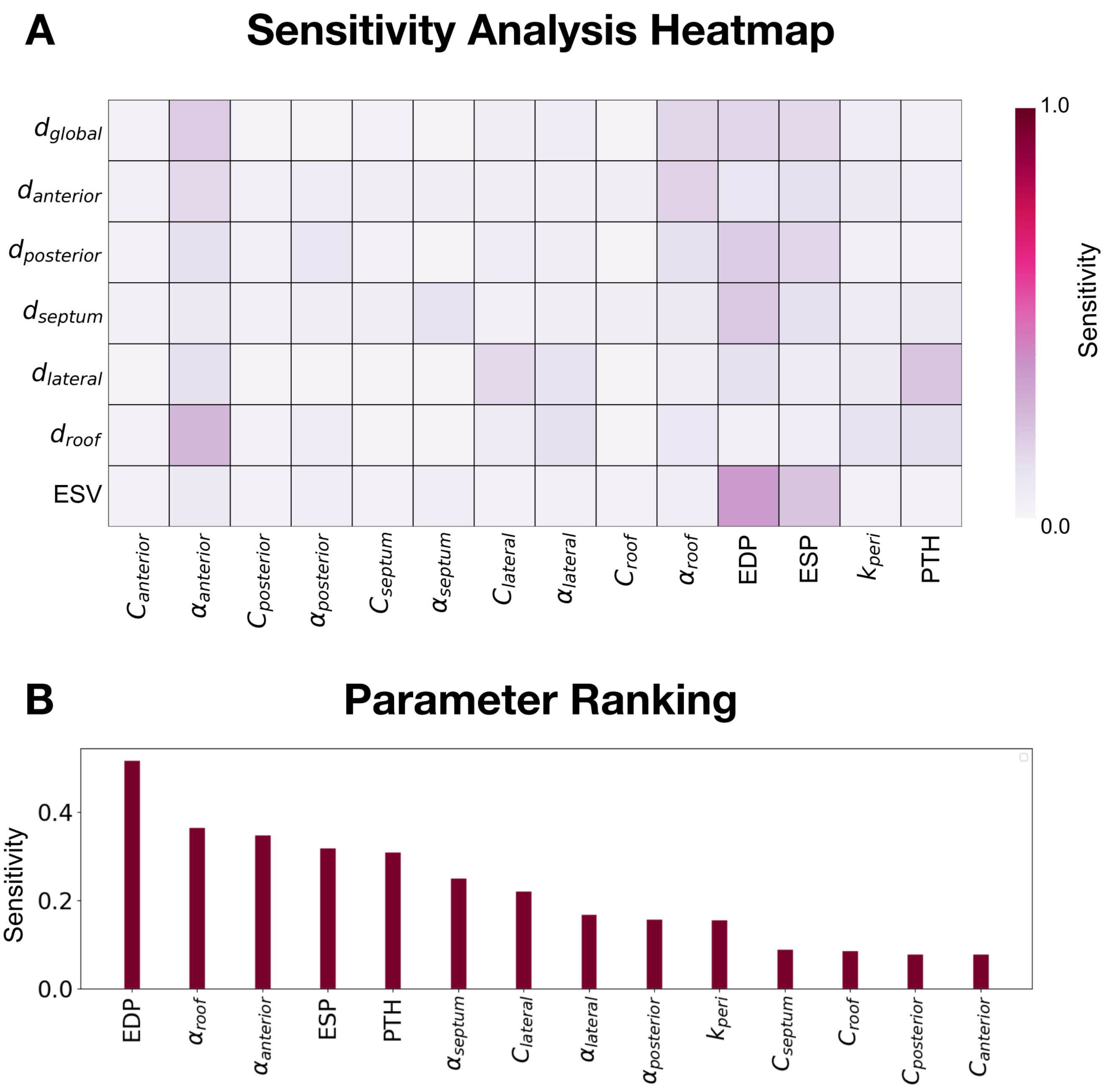

Fig 2: **Global sensitivity analysis results for case 01**. **A** Heatmap of the total effect of the parameters (x-axis) on the outputs (y-axis). **B** Barplot of the maximum total effect of each parameter over all outputs. The parameters are ranked from most to least important.

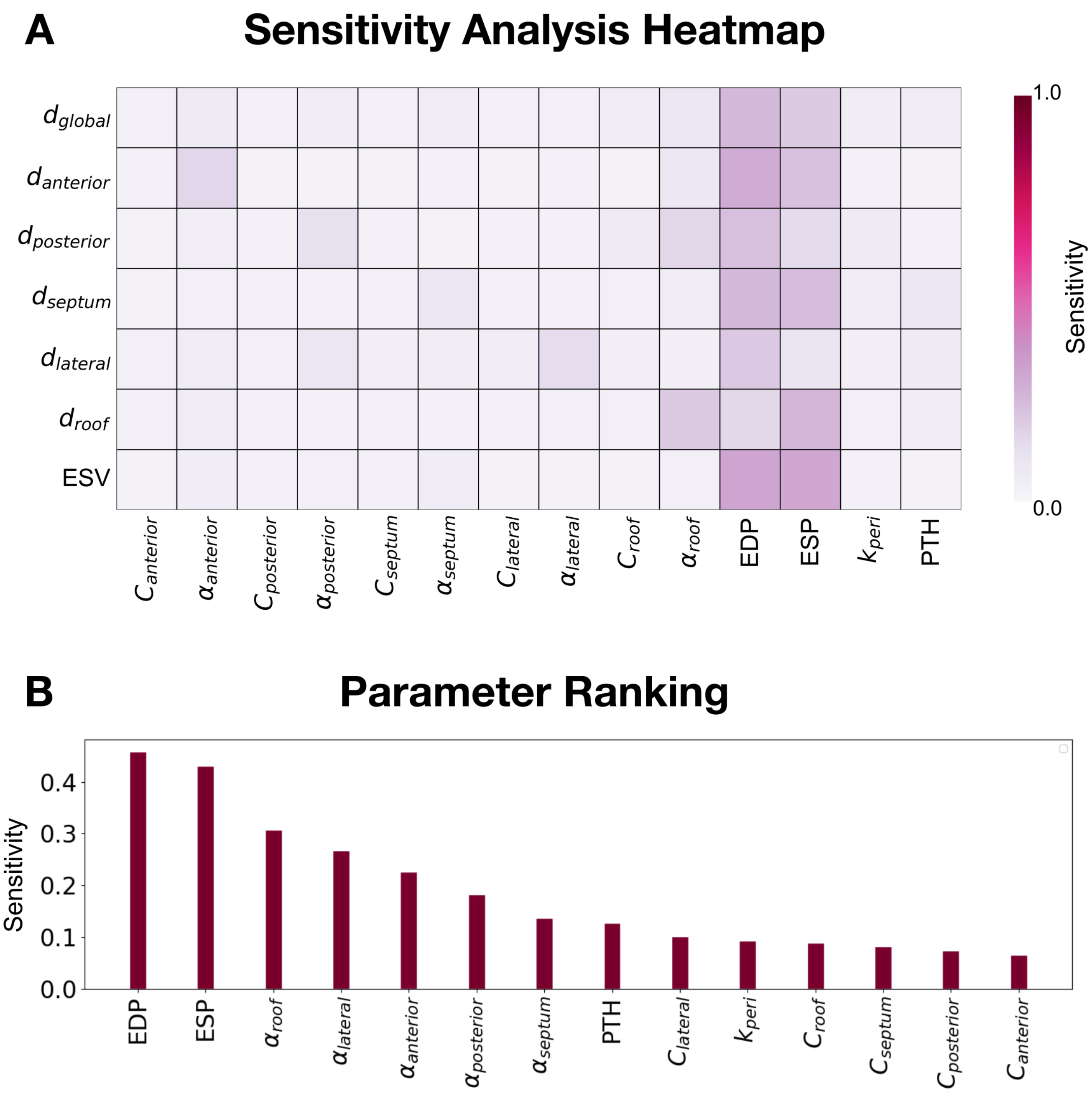

Fig 3: **Global sensitivity analysis results for case 02**. **A** Heatmap of the total effect of the parameters (x-axis) on the outputs (y-axis). **B** Barplot of the maximum total effect of each parameter over all outputs. The parameters are ranked from most to least important.

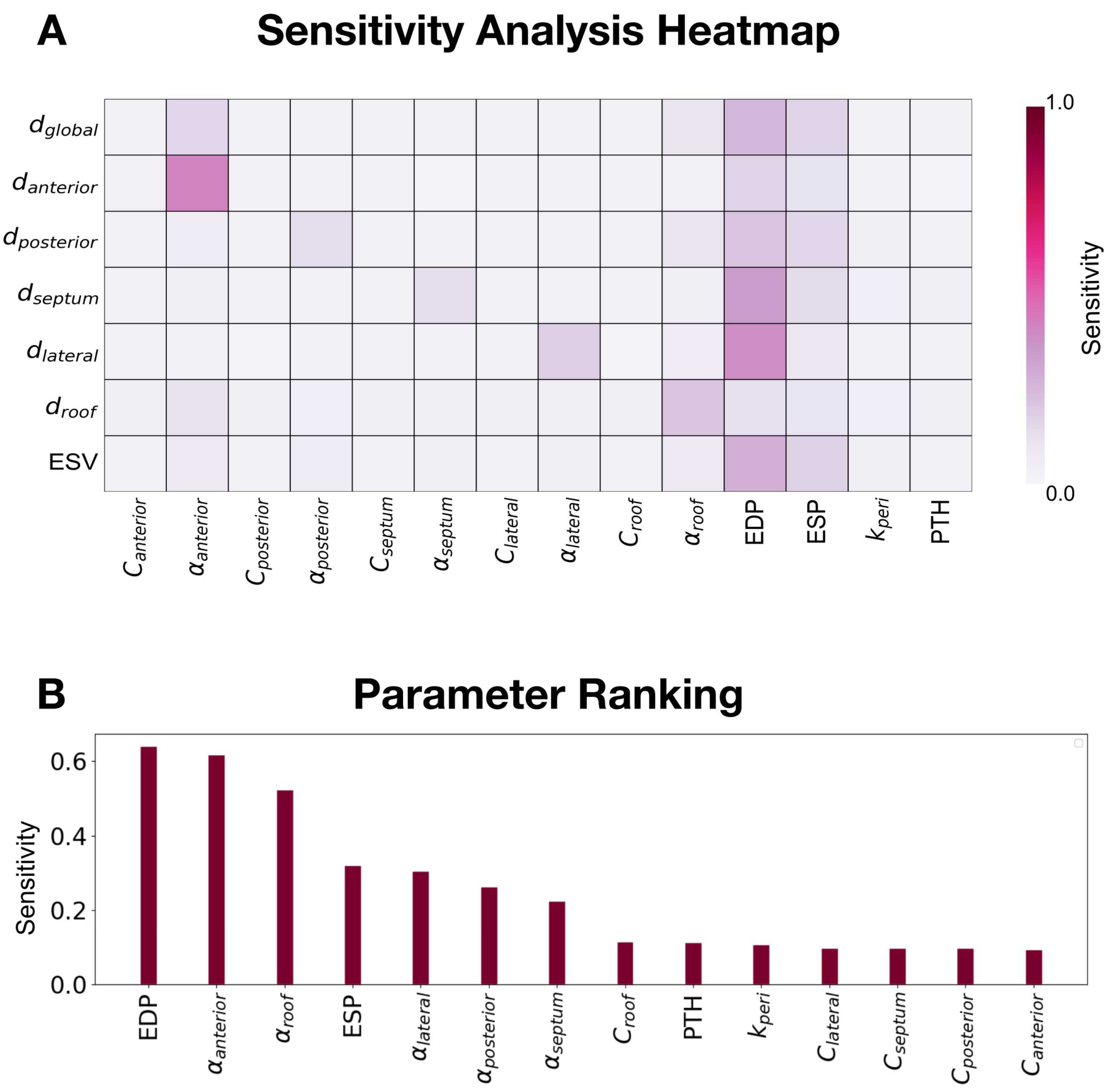

Fig 4: **Global sensitivity analysis results for case 03**. **A** Heatmap of the total effect of the parameters (x-axis) on the outputs (y-axis). **B** Barplot of the maximum total effect of each parameter over all outputs. The parameters are ranked from most to least important.

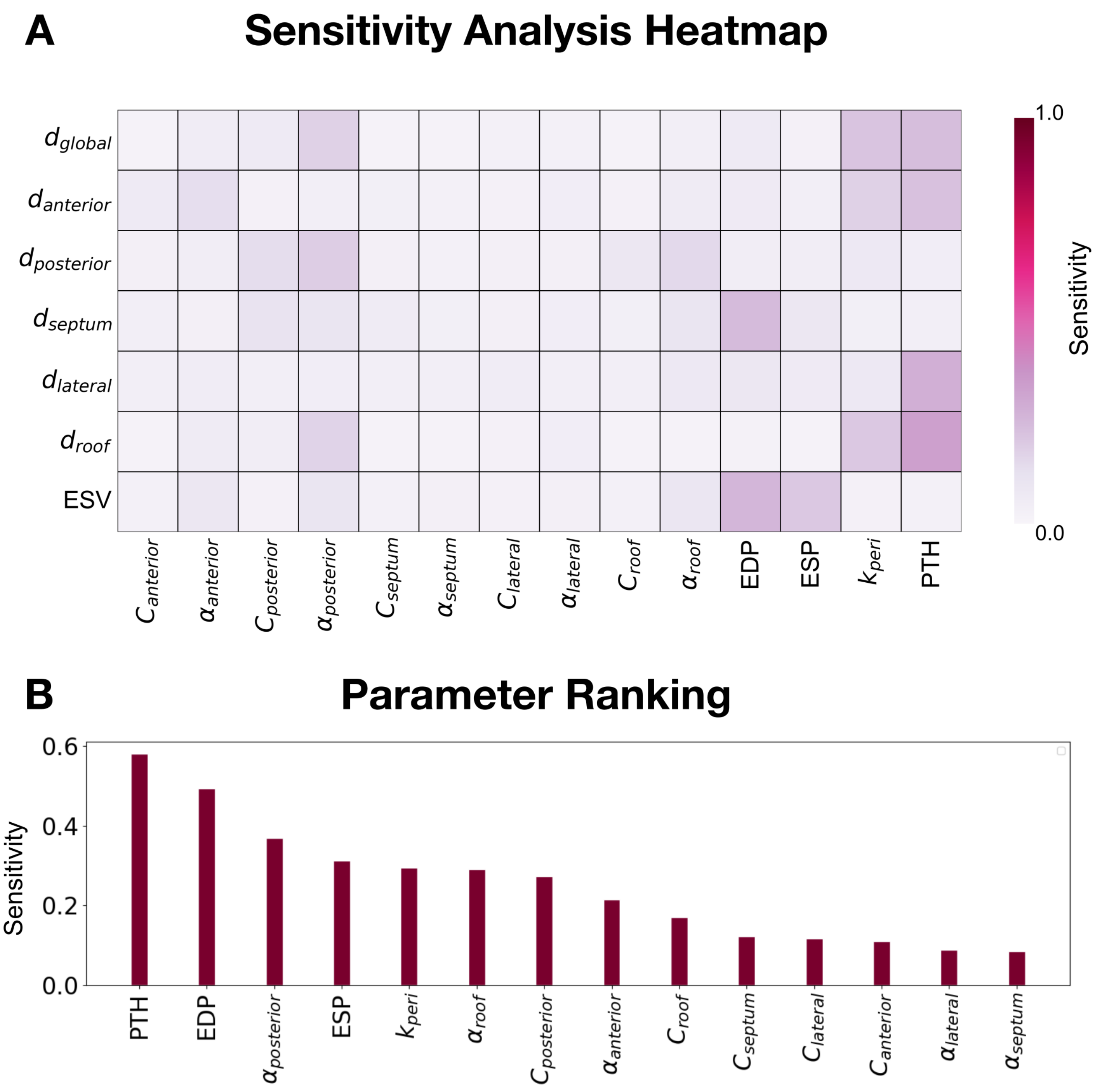

Fig 5: **Global sensitivity analysis results for case 04**. **A** Heatmap of the total effect of the parameters (x-axis) on the outputs (y-axis). **B** Barplot of the maximum total effect of each parameter over all outputs. The parameters are ranked from most to least important.

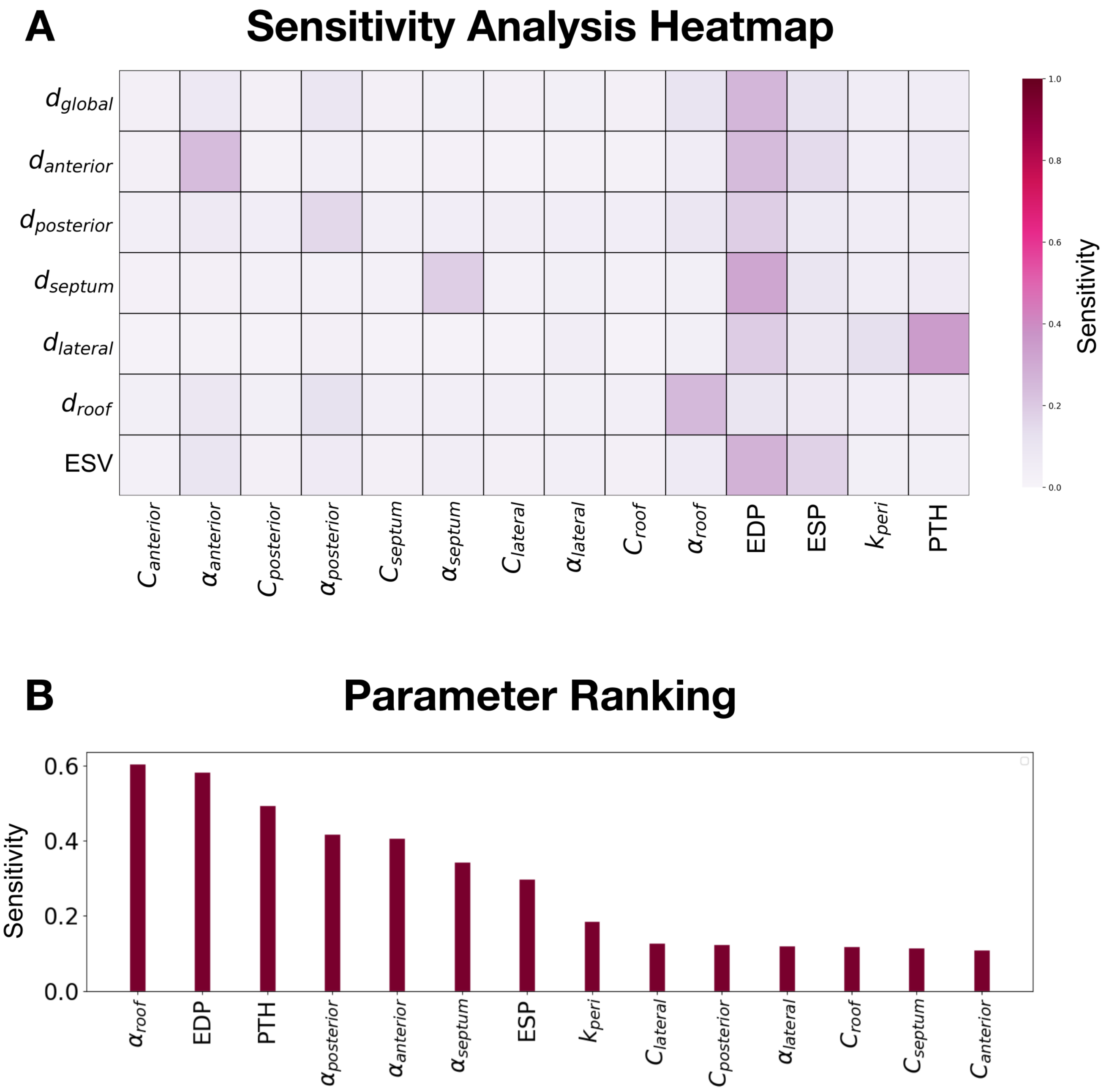

Fig 6: **Global sensitivity analysis results for case 05**. **A** Heatmap of the total effect of the parameters (x-axis) on the outputs (y-axis). **B** Barplot of the maximum total effect of each parameter over all outputs. The parameters are ranked from most to least important.

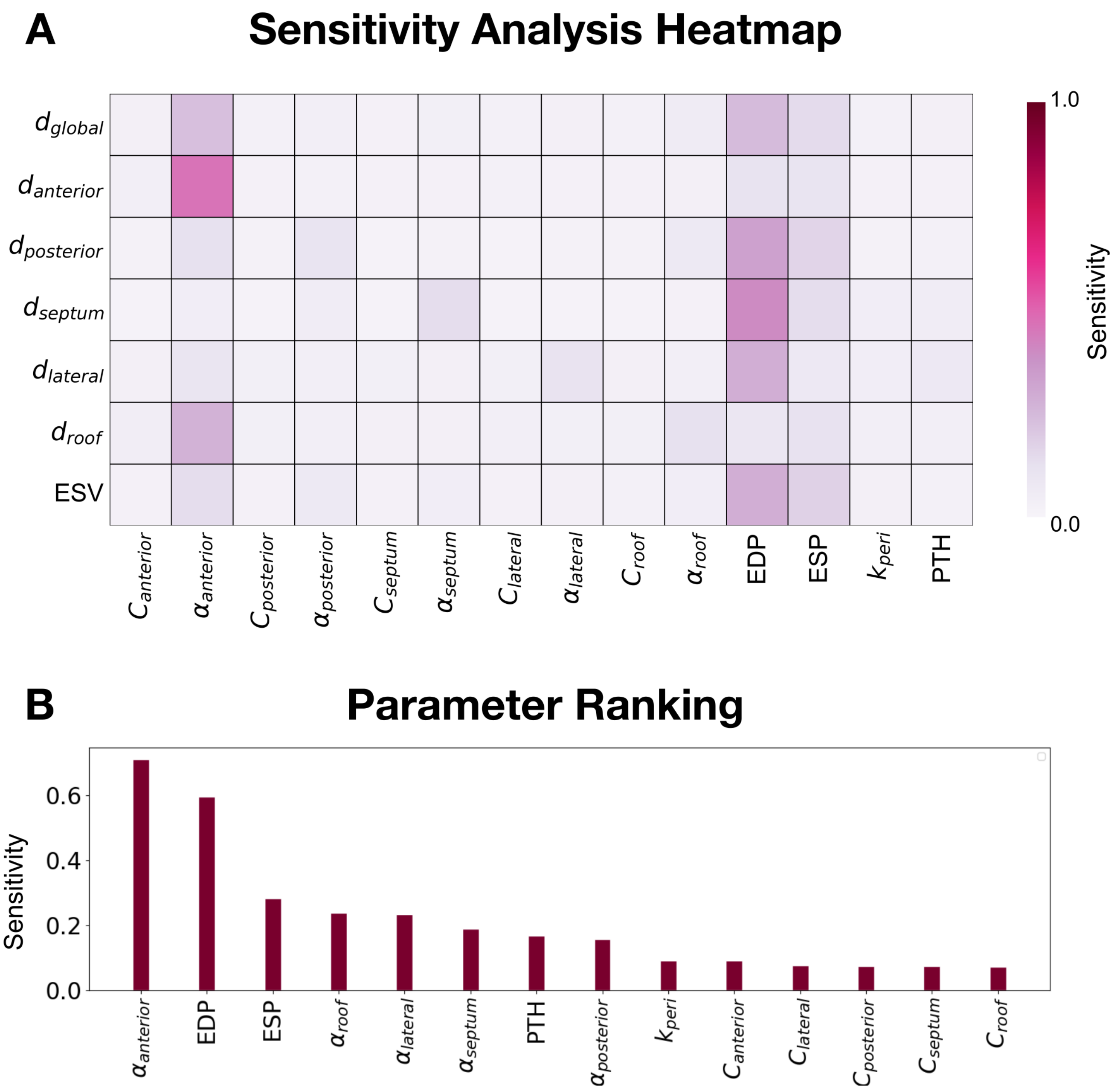

Fig 7: **Global sensitivity analysis results for case 06**. **A** Heatmap of the total effect of the parameters (x-axis) on the outputs (y-axis). **B** Barplot of the maximum total effect of each parameter over all outputs. The parameters are ranked from most to least important.

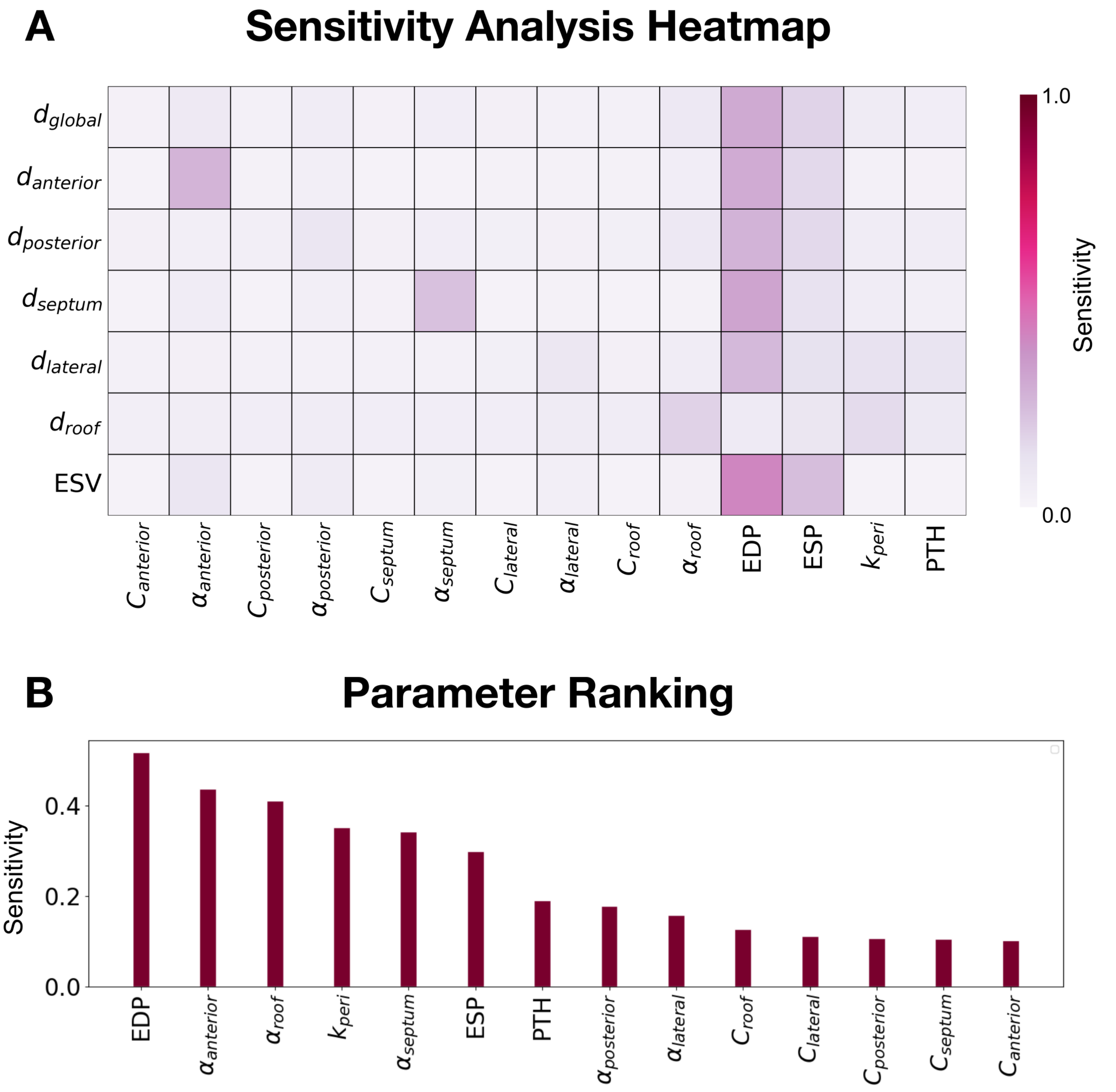

Fig 8: **Global sensitivity analysis results for case 07**. **A** Heatmap of the total effect of the parameters (x-axis) on the outputs (y-axis). **B** Barplot of the maximum total effect of each parameter over all outputs. The parameters are ranked from most to least important.

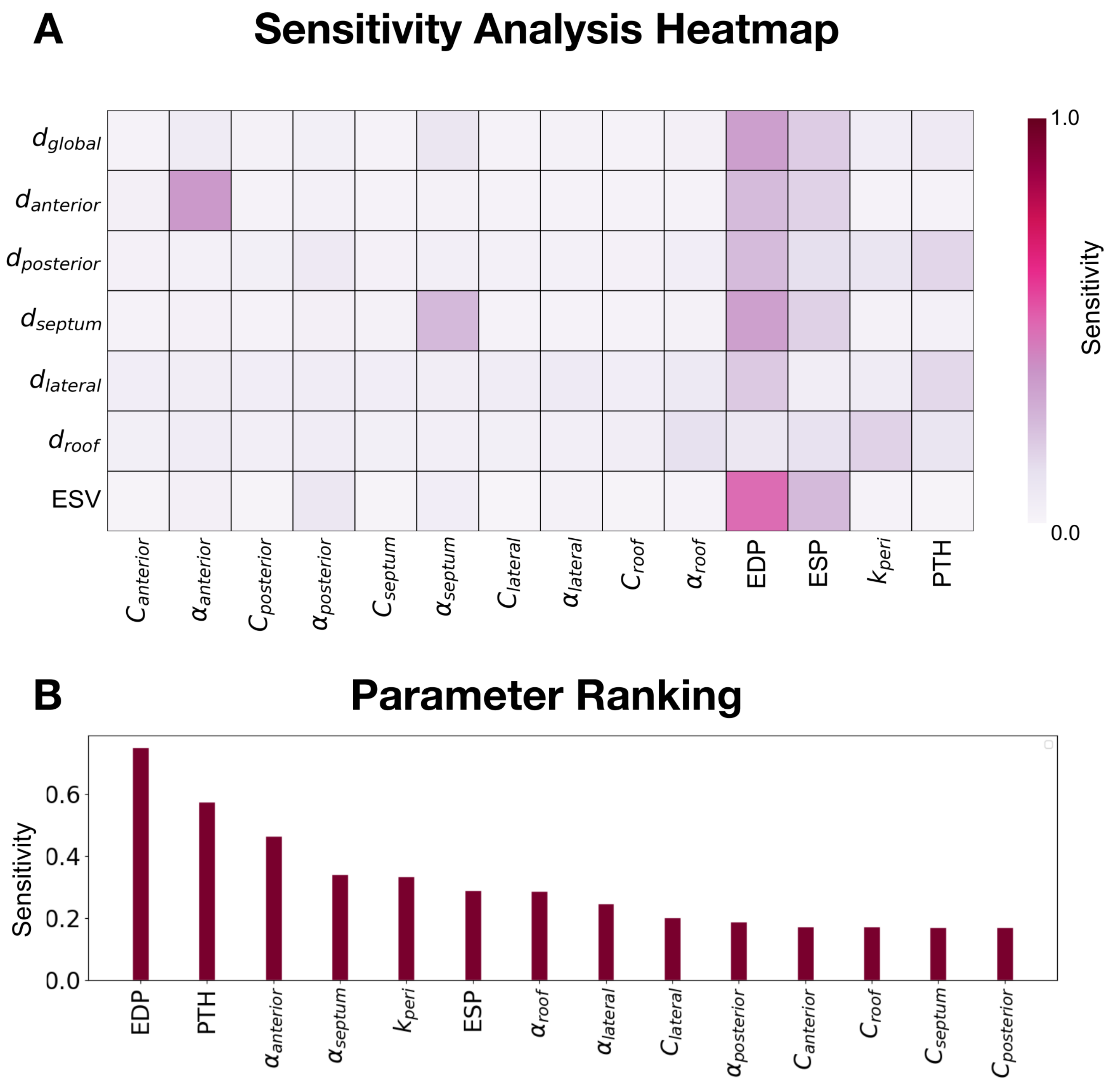

Fig 9: **Global sensitivity analysis results for case 08**. **A** Heatmap of the total effect of the parameters (x-axis) on the outputs (y-axis). **B** Barplot of the maximum total effect of each parameter over all outputs. The parameters are ranked from most to least important.

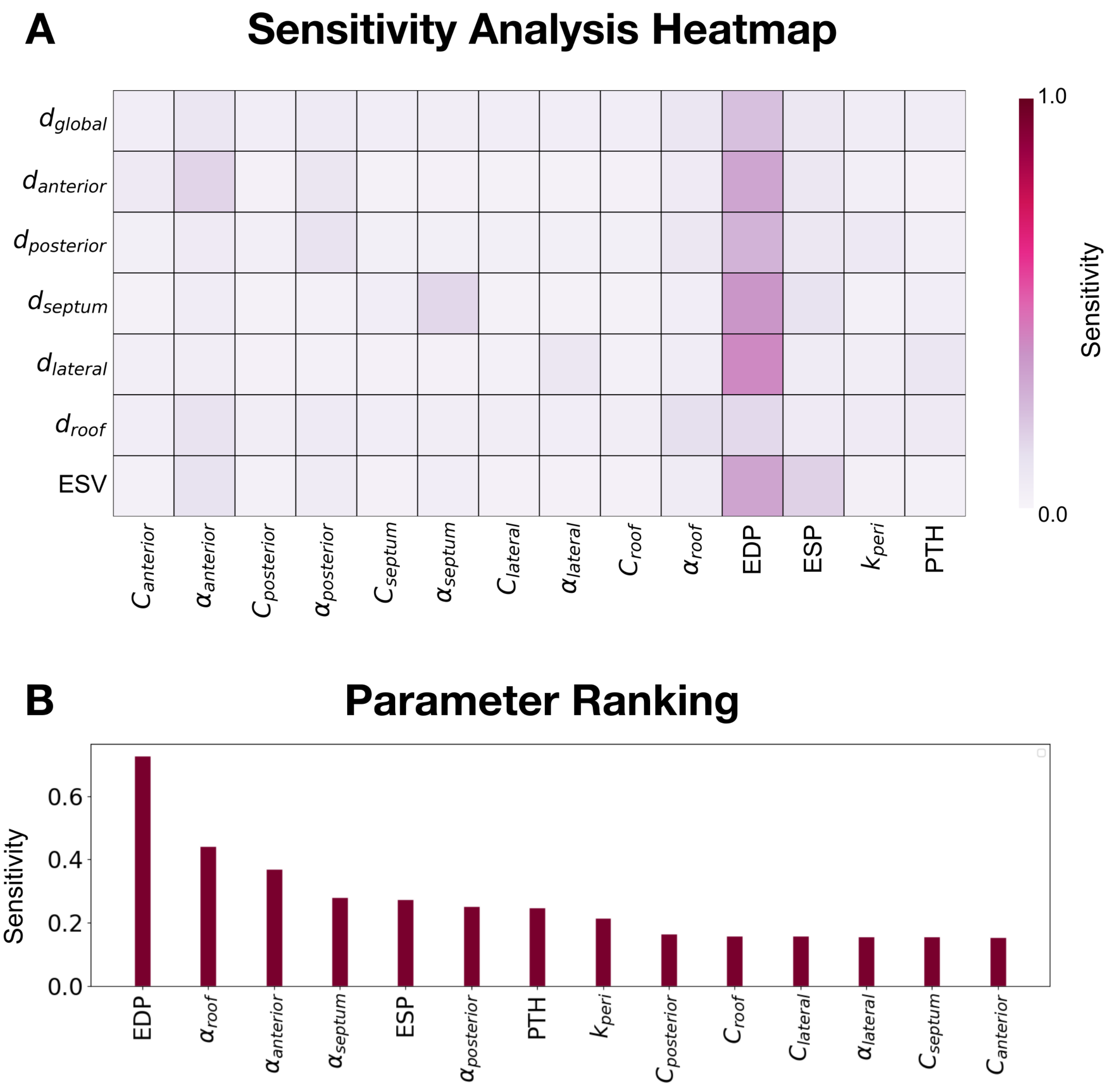

Fig 10: **Global sensitivity analysis results for case 09**. **A** Heatmap of the total effect of the parameters (x-axis) on the outputs (y-axis). **B** Barplot of the maximum total effect of each parameter over all outputs. The parameters are ranked from most to least important.

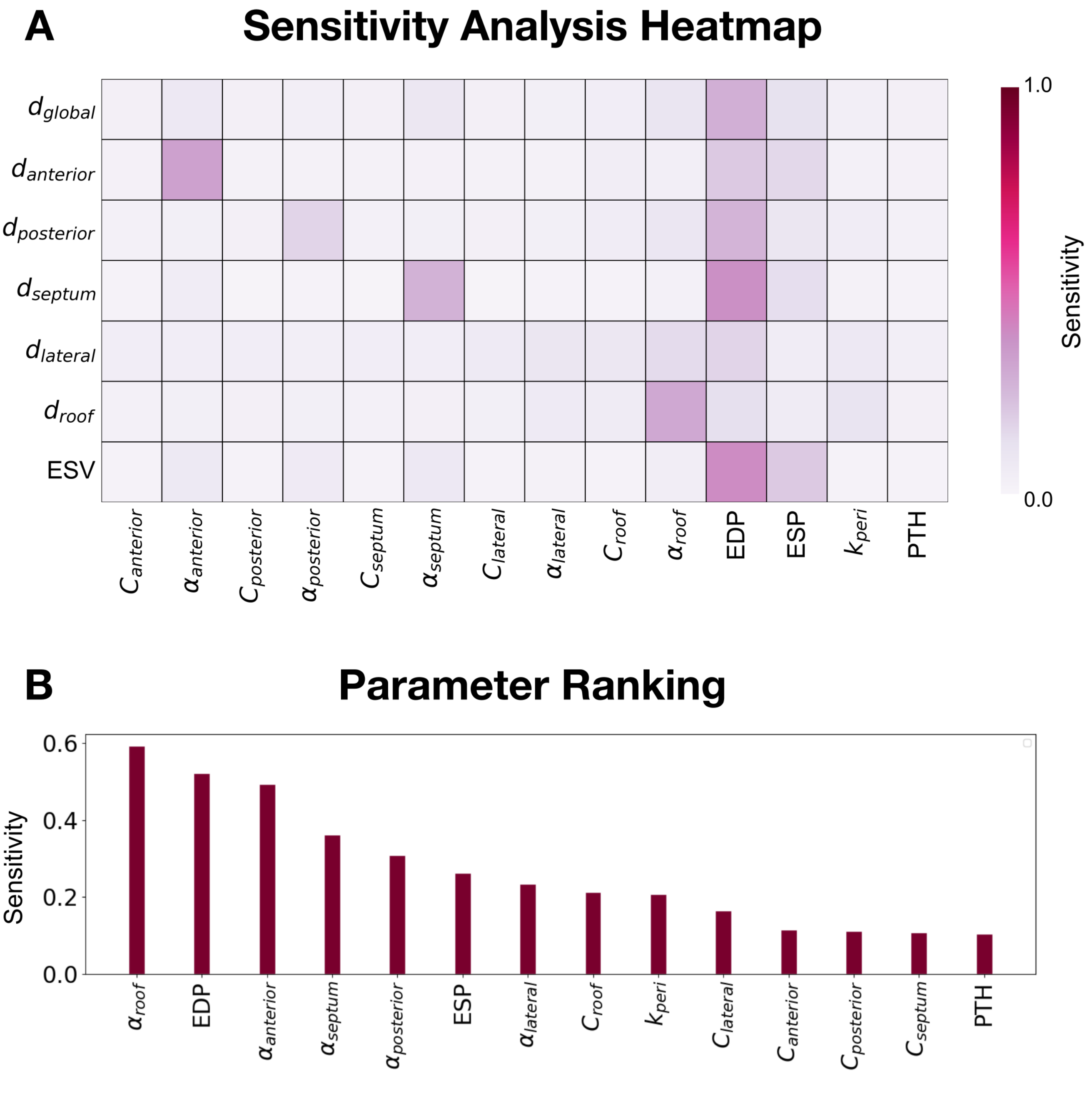

Fig 11: **Global sensitivity analysis results for case 10**. **A** Heatmap of the total effect of the parameters (x-axis) on the outputs (y-axis). **B** Barplot of the maximum total effect of each parameter over all outputs. The parameters are ranked from most to least important.

References

1. Sobol IM. Global sensitivity indices for nonlinear mathematical models and their Monte Carlo estimates. Mathematics and Computers in Simulation. 2001;55(1-3):271–280. doi:10.1016/S0378-4754(00)00270-6.

2. Saltelli A, Annoni P, Azzini I, Campolongo F, Ratto M, Tarantola S. Variance based sensitivity analysis of model output. Design and estimator for the total sensitivity index. Computer Physics Communications. 2010;181(2):259–270. doi:10.1016/J.CPC.2009.09.018.

3. Herman J, Usher W. SALib: An open-source Python library for Sensitivity Analysis. Journal of Open Source Software. 2017;2(9):97. doi:10.21105/JOSS.00097.

4. Strocchi M, Longobardi S, Augustin CM, Gsell MAF, Petras A, Rinaldi CA, et al. Cell to whole organ global sensitivity analysis on a four-chamber heart electromechanics model using Gaussian processes emulators. PLOS Computational Biology. 2023;19(6):e1011257. doi:10.1371/JOURNAL.PCBI.1011257.
