# Supplementary material for: Regional heterogeneity in left atrial stiffness impacts passive deformation in a cohort of patient-specific models
Source: PLoS Comput Biol. 2025 Nov 5;21(11):e1013656. doi: 10.1371/journal.pcbi.1013656 (PMC12599961; doi:10.1371/journal.pcbi.1013656)
Supplement: S8 File — Summary of the GPE training results for all patient cases in this study. (PDF) [file pcbi.1013656.s008.pdf]

Gaussian Process Emulation

Table 1: GPE accuracy was evaluated using five-fold cross validation. A final GPE was then trained using the full dataset. The metric scores reported were determined from the GPE trained on the full dataset following the five splits of the cross validation process. The feature labels are explained in the main text.

	Metric	case 01	case 02	case 03	case 04	case 05	case 06	case 07	case 08	case 09	case 10
d_{global}	R^2	0.95	0.97	0.94	0.87	0.87	0.93	0.93	0.93	0.78	0.90
	ISE	0.94	0.89	0.89	0.95	0.93	0.94	0.93	0.95	0.90	0.98
$d_{anterior}$	R^2	0.88	0.97	0.97	0.83	0.93	0.92	0.93	0.98	0.72	0.93
	ISE	0.89	0.92	0.96	0.98	0.95	0.92	0.94	0.94	0.93	0.95
$d_{posterior}$	R^2	0.95	0.95	0.92	0.89	0.79	0.91	0.92	0.90	0.83	0.85
	ISE	0.91	0.89	0.92	0.93	0.95	0.94	0.96	0.89	0.92	0.95
d_{septum}	R^2	0.94	0.99	0.96	0.89	0.95	0.98	0.97	0.97	0.87	0.96
	ISE	0.89	0.90	0.93	0.95	0.95	0.92	0.96	0.94	0.91	0.96
$d_{lateral}$	R^2	0.89	0.96	0.93	0.89	0.96	0.76	0.87	0.83	0.81	0.77
	ISE	0.91	0.96	0.96	0.95	0.98	0.95	0.94	0.91	0.97	0.94
d_{roof}	R^2	0.92	0.89	0.87	0.90	0.82	0.75	0.89	0.93	0.72	0.92
	ISE	0.89	0.89	0.92	0.93	0.91	0.97	0.95	0.95	0.92	0.98
ESV	R^2	0.97	0.99	0.98	0.94	0.96	0.95	0.98	0.98	0.96	0.96
	ISE	0.95	0.94	0.94	0.93	0.95	0.94	0.95	0.94	0.95	0.97
