# Supplementary material for: Regional heterogeneity in left atrial stiffness impacts passive deformation in a cohort of patient-specific models
Source: PLoS Comput Biol. 2025 Nov 5;21(11):e1013656. doi: 10.1371/journal.pcbi.1013656 (PMC12599961; doi:10.1371/journal.pcbi.1013656)
Supplement: S9 File — Summary of the convergence of the NROY space for all patient cases in this study. (PDF) [file pcbi.1013656.s009.pdf]

History Matching

Table 1 shows the progressive reduction of the NROY space with each HM wave for each patient case. In each case, HM continued until there was a cumulative reduction of the NROY space less than 1% between successive waves.

Table 1: Implausibility cut-off threshold and size of NROY space at each wave of history matching

Wave	$I_{threshold}$	NROY (% of initial input space)									
		case 01	case 02	case 03	case 04	case 05	case 06	case 07	case 08	case 09	case 10
1	3.5	7.01	49.80	14.76	19.85	82.91	18.01	33.25	3.95	41.07	12.93
2	3	1.36	25.03	4.27	6.01	64.12	0.20	22.17	1.12	6.49	4.23
3	3	0.89	15.54	3.29	5.19	59.96	0.09	20.30	0.92	3.53	3.41
4	3	-	13.54	1.00	-	57.27	-	19.64	-	2.83	2.62
5	3	-	12.11	0.77	-	55.60	-	-	-	-	-
6	3	-	8.39	-	-	55.05	-	-	-	-	-
7	3	-	7.08	-	-	-	-	-	-	-	-
8	3	-	6.51	-	-	-	-	-	-	-	-
