# Supplementary material for: Regional heterogeneity in left atrial stiffness impacts passive deformation in a cohort of patient-specific models
Source: PLoS Comput Biol. 2025 Nov 5;21(11):e1013656. doi: 10.1371/journal.pcbi.1013656 (PMC12599961; doi:10.1371/journal.pcbi.1013656)
Supplement: S10 File — Summary of the final calibration results using MCMC for each of the 10 patient cases. (PDF) [file pcbi.1013656.s010.pdf]

Calibration results using MCMC

MCMC was used to identify a set of input parameter values that had the highest likelihood of reproducing the image-derived displacements and volumes for each case, MAP. Using the MAP for each case, we found the nearest neighbour within the final wave of HM parameter set for each case. We used those simulations to assess the calibration process accuracy. The figures below show the calibrated simulation features against the CT-derived targets for each case.

Each simulation requires the solution a series of nonlinear equations to solve a large deformation mechanics problem using the finite element method. The complex geometry of the heart and its individual chambers lead to a well-recognised issue of non-convergence in cardiac mechanics models [1]. The heterogeneous LA geometry and its high variability over the patient cohort lead to issues with simulation convergence in some cases and both reservoir and conduit phases were not fully simulated. Each simulations requires $\approx$ 53 cores/hour. Thus, to maximise effective use of computational resource, simulations that did not converge were still used as long as the simulation passed the ES time point as exhibited in Fig 5.

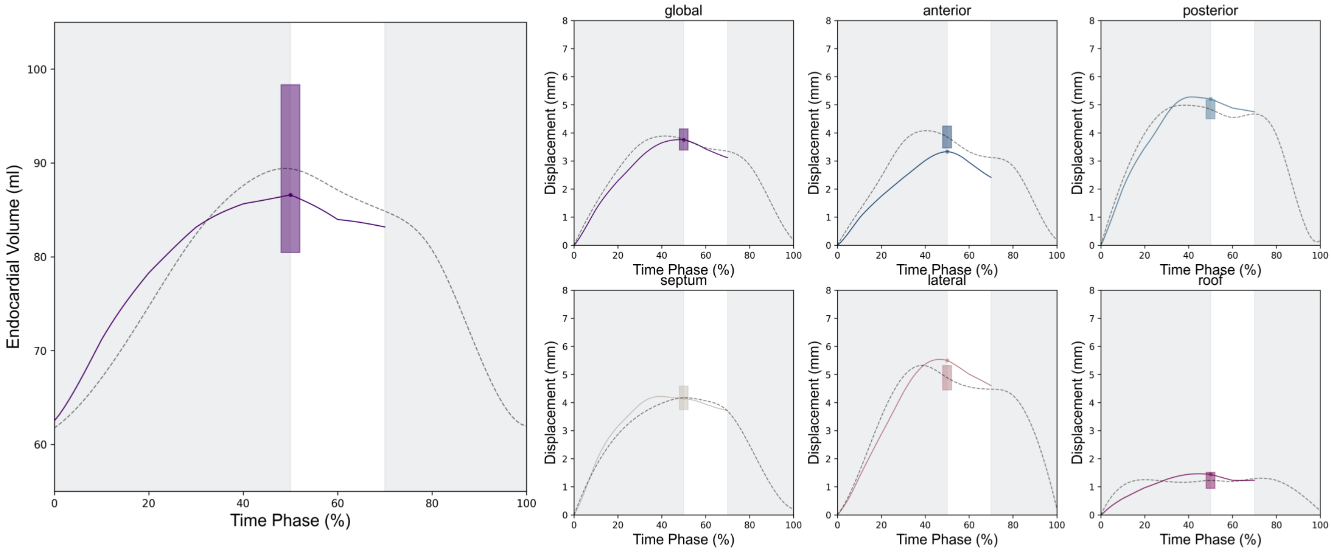

Fig 1: **Calibrated simulation results for case 01**.The panels show the global and regional displacements as well as the volumes obtained when the simulator was evaluated using the nearest neighbour of the MAP estimate. The dashed black line indicates the LA behaviour derived from the CT image set and the shaded box represents ± 2SD of the target value. The LA volume transient was used to identify the LA reservoir, conduit and booster pump phases.

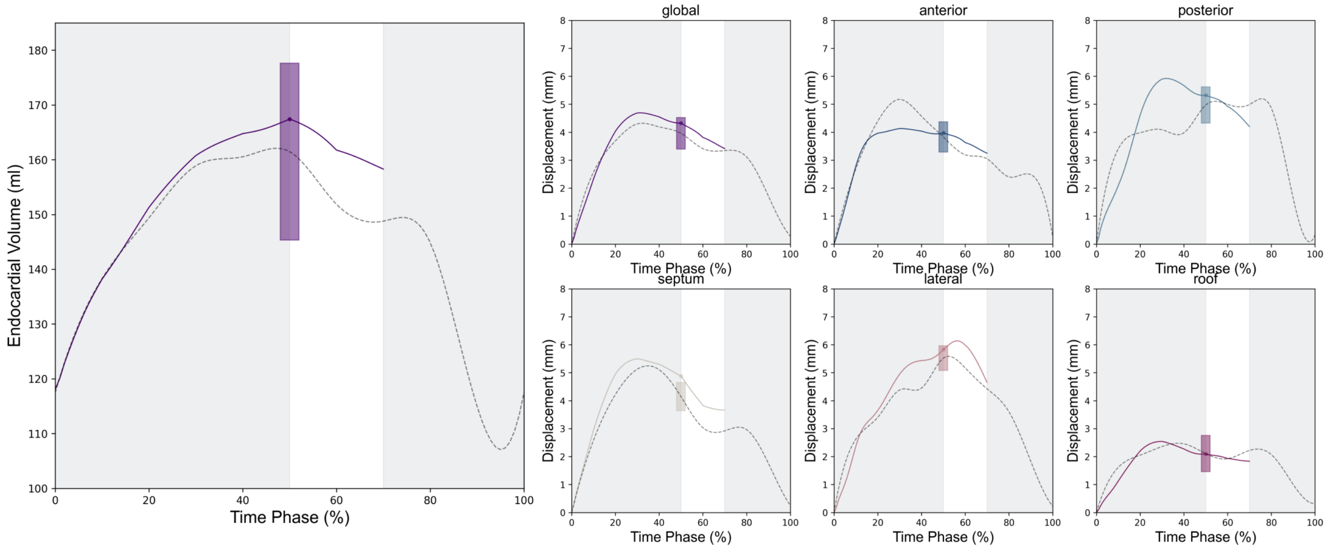

Fig 2: **Calibrated simulation results for case 02**.The panels show the global and regional displacements as well as the volumes obtained when the simulator was evaluated using the nearest neighbour of the MAP estimate. The dashed black line indicates the LA behaviour derived from the CT image set and the shaded box represents ± 2SD of the target value. The LA volume transient was used to identify the LA reservoir, conduit and booster pump phases.

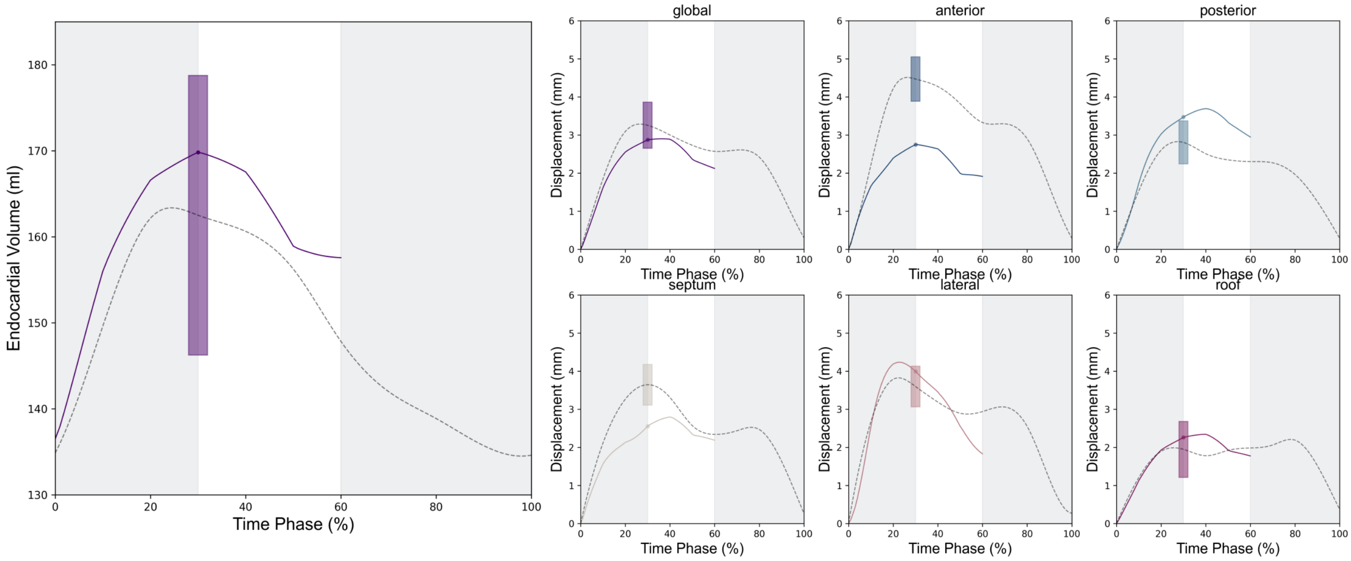

Fig 3: **Calibrated simulation results for case 03**.The panels show the global and regional displacements as well as the volumes obtained when the simulator was evaluated using the nearest neighbour of the MAP estimate. The dashed black line indicates the LA behaviour derived from the CT image set and the shaded box represents ± 2SD of the target value. The LA volume transient was used to identify the LA reservoir, conduit and booster pump phases.

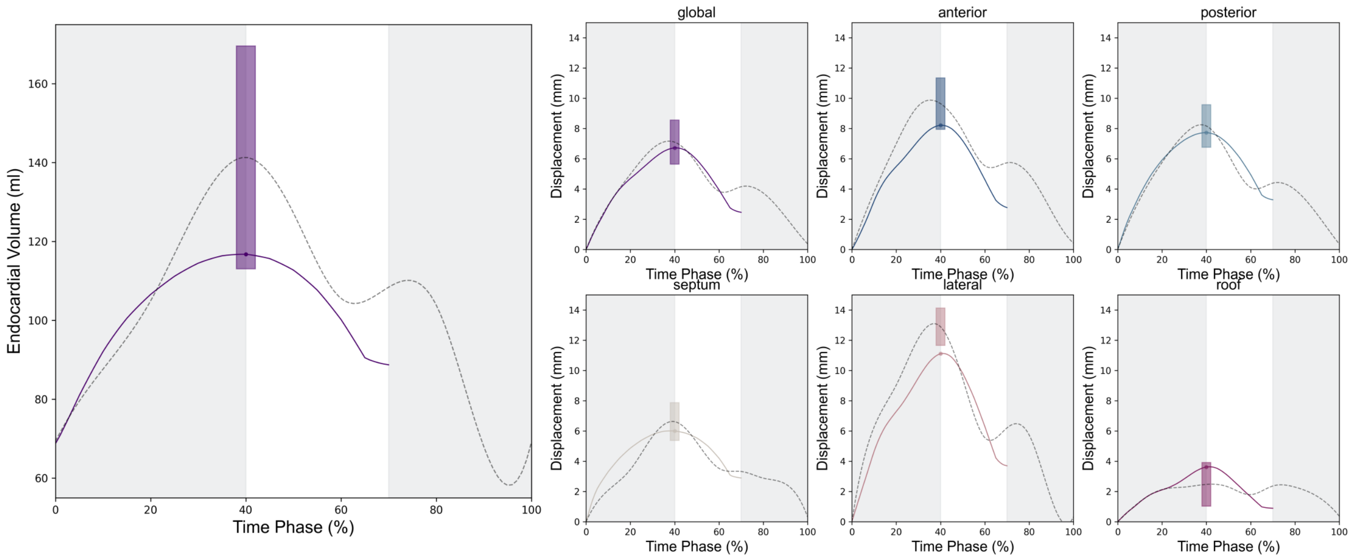

Fig 4: **Calibrated simulation results for case 04**.The panels show the global and regional displacements as well as the volumes obtained when the simulator was evaluated using the nearest neighbour of the MAP estimate. The dashed black line indicates the LA behaviour derived from the CT image set and the shaded box represents ± 2SD of the target value. The LA volume transient was used to identify the LA reservoir, conduit and booster pump phases.

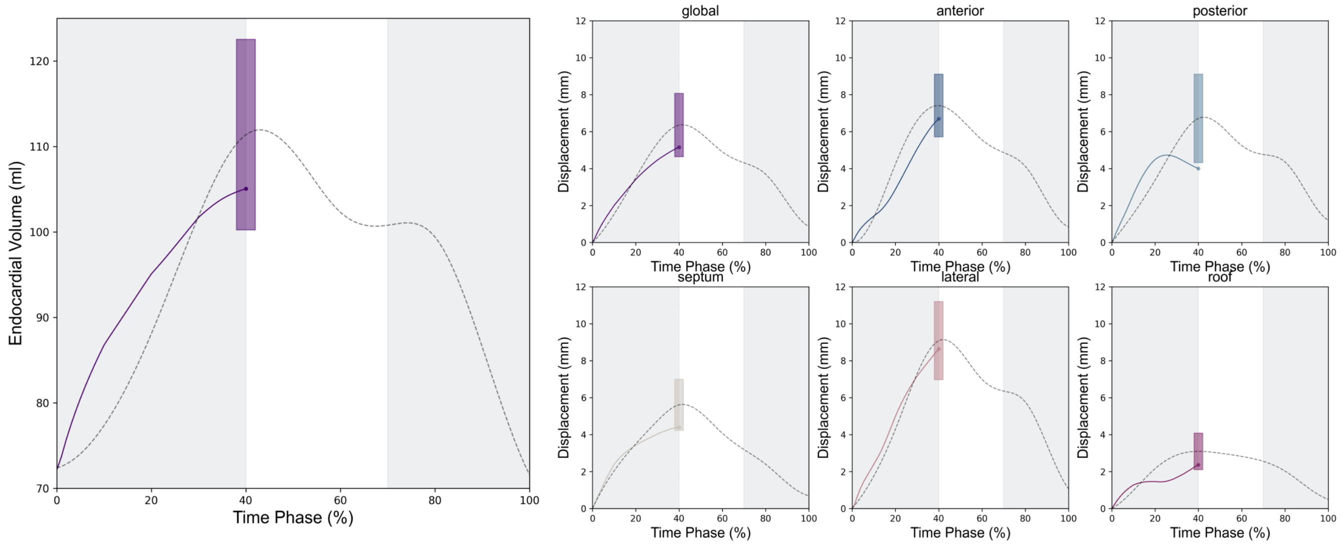

Fig 5: **Calibrated simulation results for case 05**.The panels show the global and regional displacements as well as the volumes obtained when the simulator was evaluated using the nearest neighbour of the MAP estimate. The dashed black line indicates the LA behaviour derived from the CT image set and the shaded box represents ± 2SD of the target value. The LA volume transient was used to identify the LA reservoir, conduit and booster pump phases.

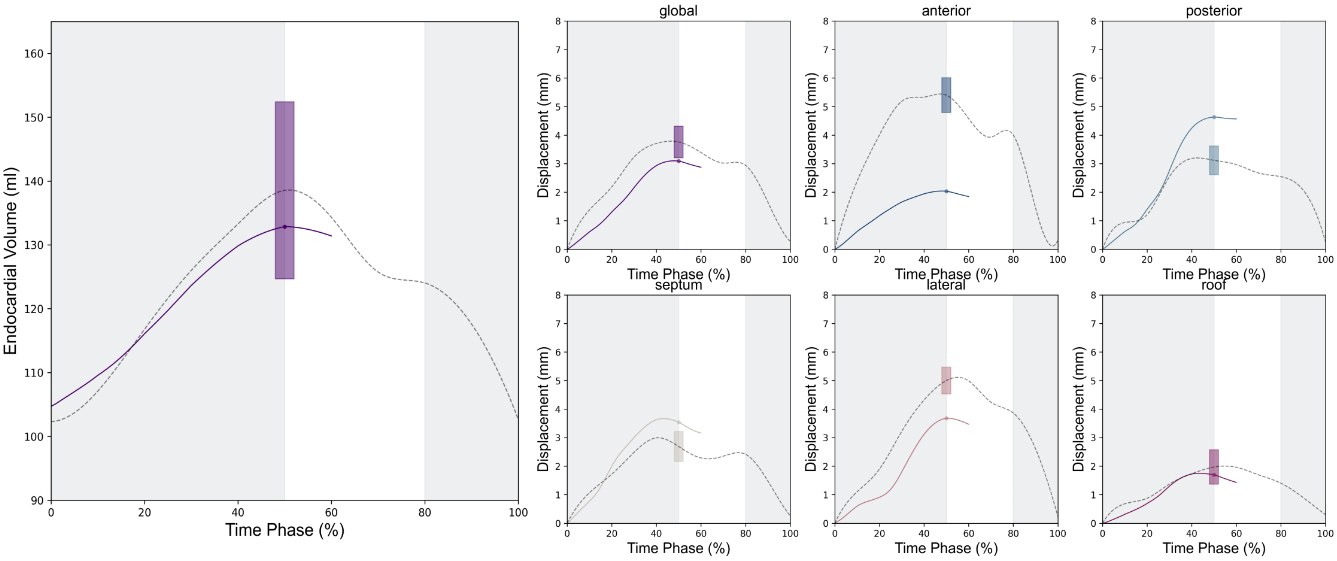

Fig 6: **Calibrated simulation results for case 06**.The panels show the global and regional displacements as well as the volumes obtained when the simulator was evaluated using the nearest neighbour of the MAP estimate. The dashed black line indicates the LA behaviour derived from the CT image set and the shaded box represents ± 2SD of the target value. The LA volume transient was used to identify the LA reservoir, conduit and booster pump phases.

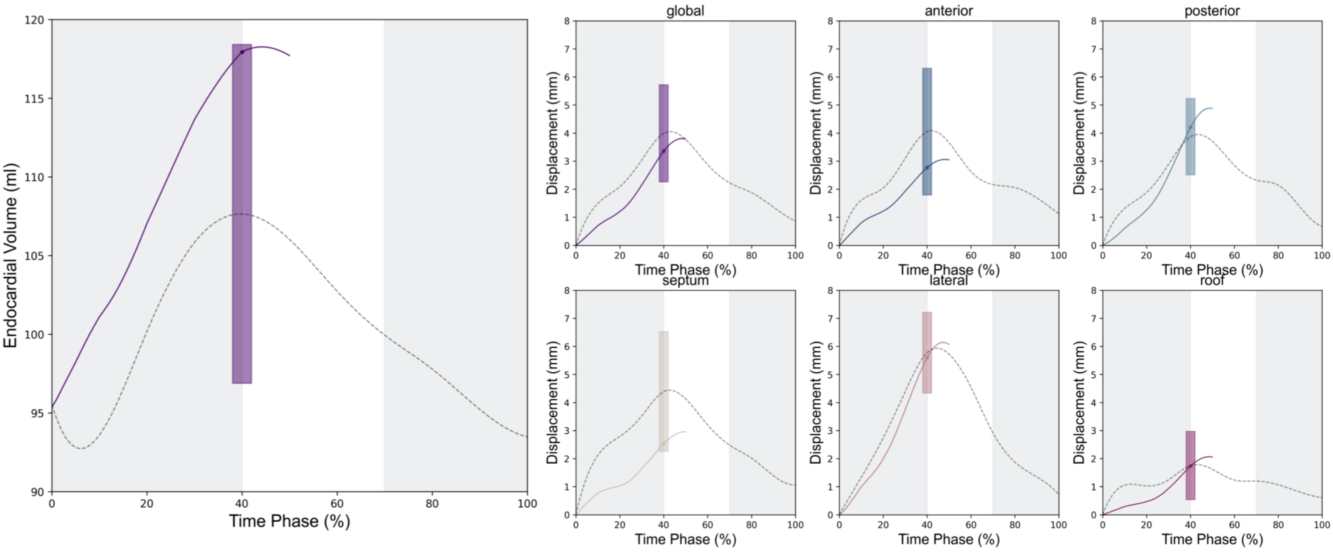

Fig 7: **Calibrated simulation results for case 07**.The panels show the global and regional displacements as well as the volumes obtained when the simulator was evaluated using the nearest neighbour of the MAP estimate. The dashed black line indicates the LA behaviour derived from the CT image set and the shaded box represents ± 2SD of the target value. The LA volume transient was used to identify the LA reservoir, conduit and booster pump phases.

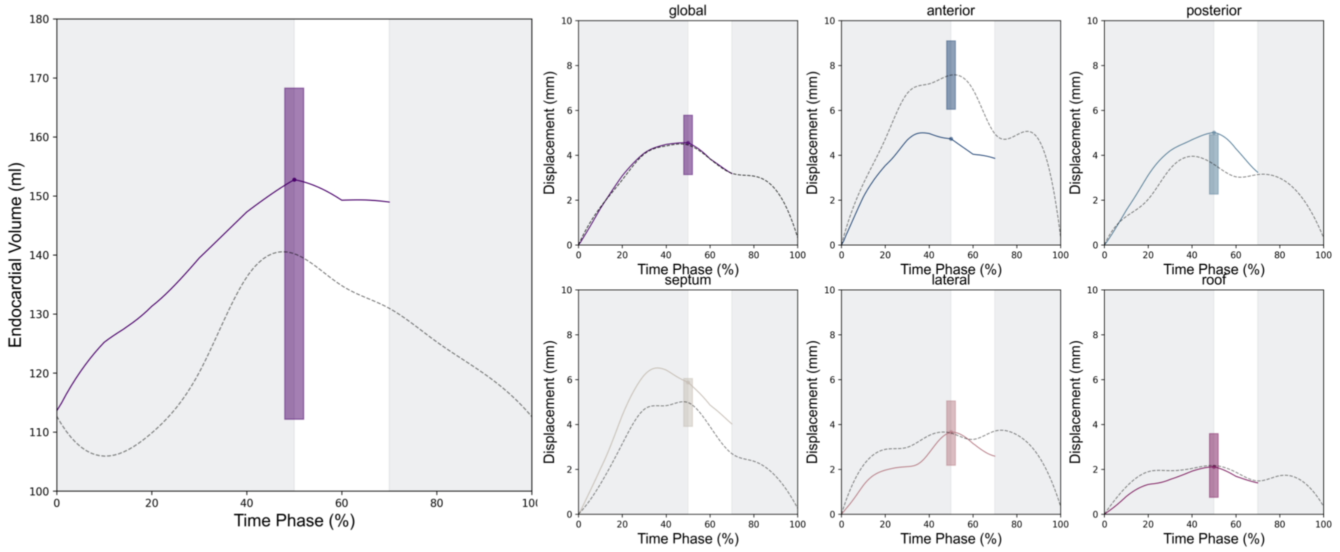

Fig 8: **Calibrated simulation results for case 08**.The panels show the global and regional displacements as well as the volumes obtained when the simulator was evaluated using the nearest neighbour of the MAP estimate. The dashed black line indicates the LA behaviour derived from the CT image set and the shaded box represents ± 2SD of the target value. The LA volume transient was used to identify the LA reservoir, conduit and booster pump phases.

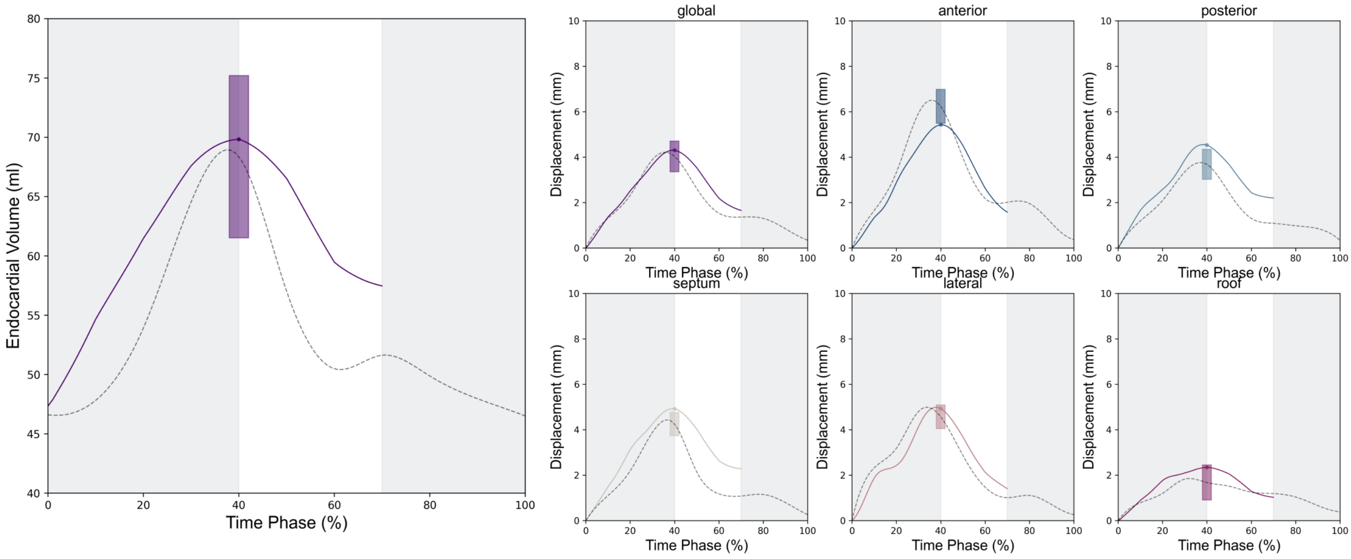

Fig 9: **Calibrated simulation results for case 09**.The panels show the global and regional displacements as well as the volumes obtained when the simulator was evaluated using the nearest neighbour of the MAP estimate. The dashed black line indicates the LA behaviour derived from the CT image set and the shaded box represents ± 2SD of the target value. The LA volume transient was used to identify the LA reservoir, conduit and booster pump phases.

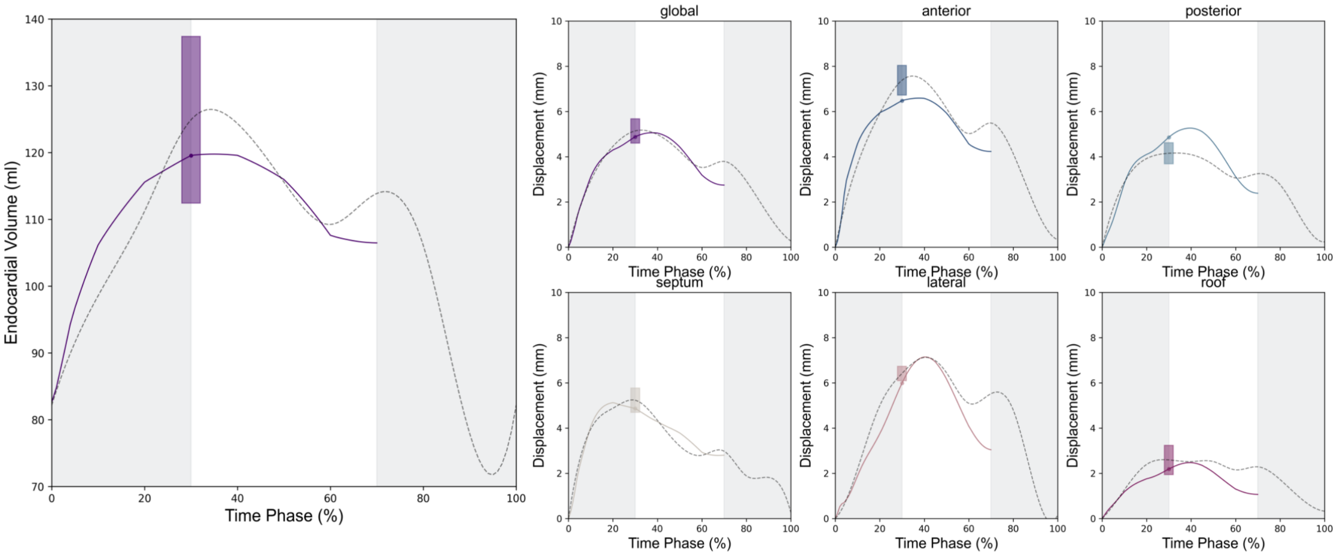

Fig 10: **Calibrated simulation results for case 10**.The panels show the global and regional displacements as well as the volumes obtained when the simulator was evaluated using the nearest neighbour of the MAP estimate. The dashed black line indicates the LA behaviour derived from the CT image set and the shaded box represents ± 2SD of the target value. The LA volume transient was used to identify the LA reservoir, conduit and booster pump phases.

The regional calibrated stiffness parameters for each patient case obtained using MCMC are shown in table 1

Table 1: Calibrated Guccione stiffness parameters from MCMC for a cohort of HF patients

case	region	α	C	b_f	b_{ft}	b_t
1	anterior	3.590774	1.7	28.72619	14.3631	10.77232
	posteror	0.429754	1.7	3.438032	1.719016	1.289262
	septum	1.345023	1.7	10.76018	5.380092	4.035069
	lateral	0.175312	1.7	1.402496	0.701248	0.525936
	roof	0.666557	1.7	5.332456	2.666228	1.999671
2	anterior	1.373769	1.7	10.99015	5.495076	4.121307
	posteror	1.360987	1.7	10.8879	5.443948	4.082961
	septum	3.819988	1.7	30.5599	15.27995	11.45996
	lateral	2.758743	1.7	22.06994	11.03497	8.276229
	roof	2.640653	1.7	21.12522	10.56261	7.921959
3	anterior	0.653201	1.7	5.225608	2.612804	1.959603
	posteror	2.657801	1.7	21.26241	10.6312	7.973403
	septum	1.215475	1.7	9.7238	4.8619	3.646425
	lateral	3.499022	1.7	27.99218	13.99609	10.49707
	roof	2.832328	1.7	22.65862	11.32931	8.496984
4	anterior	3.840463	1.7	30.7237	15.36185	11.52139
	posteror	0.23291	1.7	1.86328	0.93164	0.69873
	septum	2.759391	1.7	22.07513	11.03756	8.278173
	lateral	0.3456	1.7	2.7648	1.3824	1.0368
	roof	3.960189	1.7	31.68151	15.84076	11.88057
5	anterior	3.61773	1.7	28.94184	14.47092	10.85319
	posteror	1.013393	1.7	8.107144	4.053572	3.040179
	septum	3.880382	1.7	31.04306	15.52153	11.64115
	lateral	1.587864	1.7	12.70291	6.351456	4.763592
	roof	2.912314	1.7	23.29851	11.64926	8.736942
6	anterior	0.322009	1.7	2.576072	1.288036	0.966027
	posteror	0.725798	1.7	5.806384	2.903192	2.177394
	septum	2.173216	1.7	17.38573	8.692864	6.519648
	lateral	1.058458	1.7	8.467664	4.233832	3.175374
	roof	3.755245	1.7	30.04196	15.02098	11.26574
7	anterior	0.346033	1.7	2.768264	1.384132	1.038099
	posteror	3.211303	1.7	25.69042	12.84521	9.633909
	septum	0.268864	1.7	2.150912	1.075456	0.806592
	lateral	1.696894	1.7	13.57515	6.787576	5.090682
	roof	1.017488	1.7	8.139904	4.069952	3.052464
8	anterior	0.261373	1.7	2.090984	1.045492	0.784119
	posteror	1.172063	1.7	9.376504	4.688252	3.516189
	septum	3.4265	1.7	27.412	13.706	10.2795
	lateral	3.916532	1.7	31.33226	15.66613	11.7496
	roof	0.239132	1.7	1.913056	0.956528	0.717396
9	anterior	3.440884	1.7	27.52707	13.76354	10.32265
	posteror	0.917302	1.7	7.338416	3.669208	2.751906
	septum	1.81855	1.7	14.5484	7.2742	5.45565
	lateral	0.720948	1.7	5.767584	2.883792	2.162844
	roof	0.990992	1.7	7.927936	3.963968	2.972976
10	anterior	0.562765	1.7	4.50212	2.25106	1.688295
	posteror	3.987717	1.7	31.90174	15.95087	11.96315
	septum	1.60748	1.7	12.85984	6.42992	4.82244
	lateral	0.246884	1.7	1.975072	0.987536	0.740652
	roof	2.020845	1.7	16.16676	8.08338	6.062535

The posterior distribution of the input space obtained using MCMC for the 10 patient cases in our cohort are shown in the following figures.

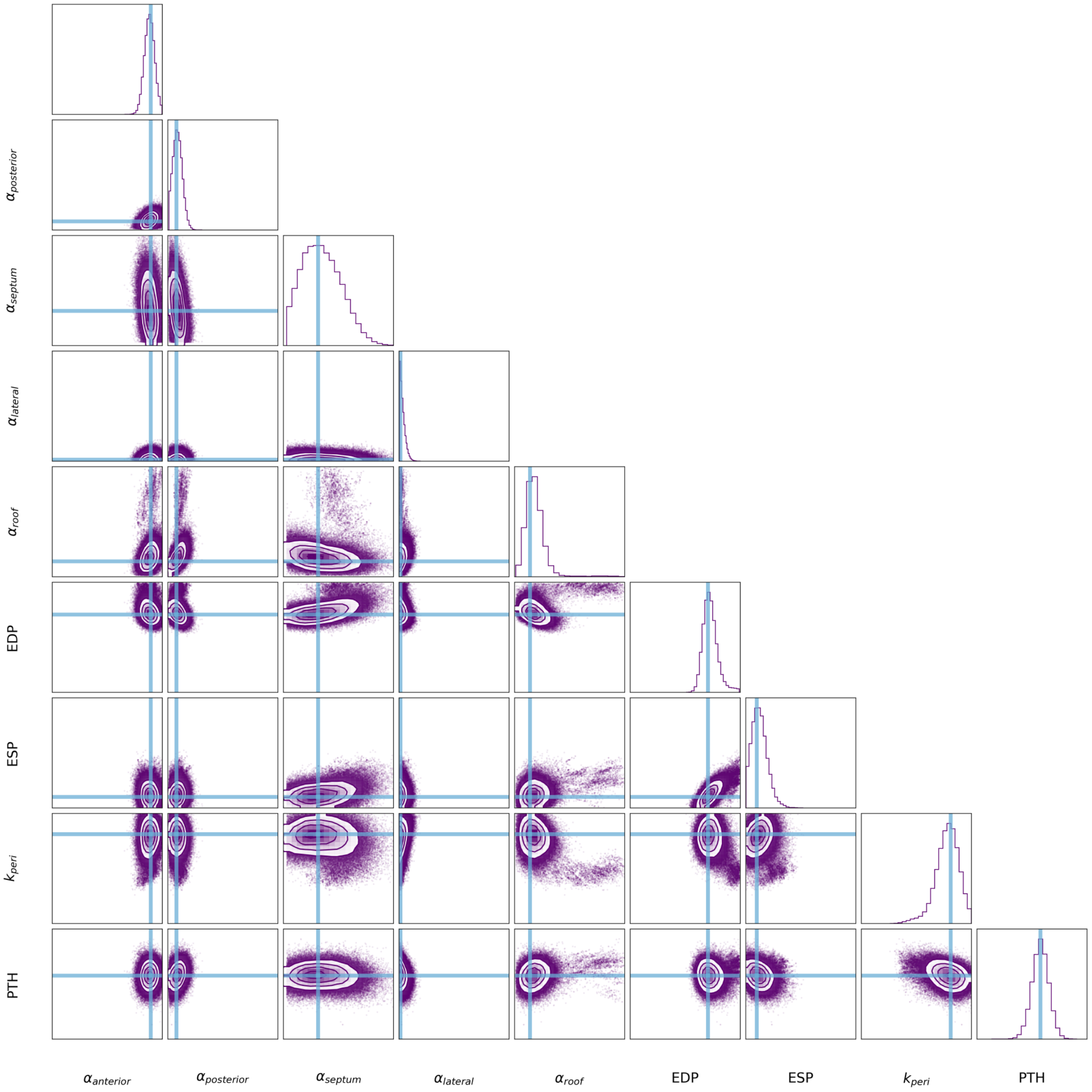

Fig 11: **Calibration using MCMC for case 01**. Plots showing the parameter distributions for case 01 estimated using MCMC. Each tile represents a projection of the Markov Chain samples. We implemented an ensemble sampler of 18 parallel walkers of 100 000 steps. A burn-in period of 10 000 steps and a thinning frequency of 10 was used. For each parameter, the MAP estimate is indicated by the blue line. The histograms along the diagonal display the posterior distributions for each parameter individually. The off-diagonal panels show the 2D distributions as contour plots where the contours are drawn at levels containing 11.8%, 39.3%, 67.5% and 86.4% of the samples.

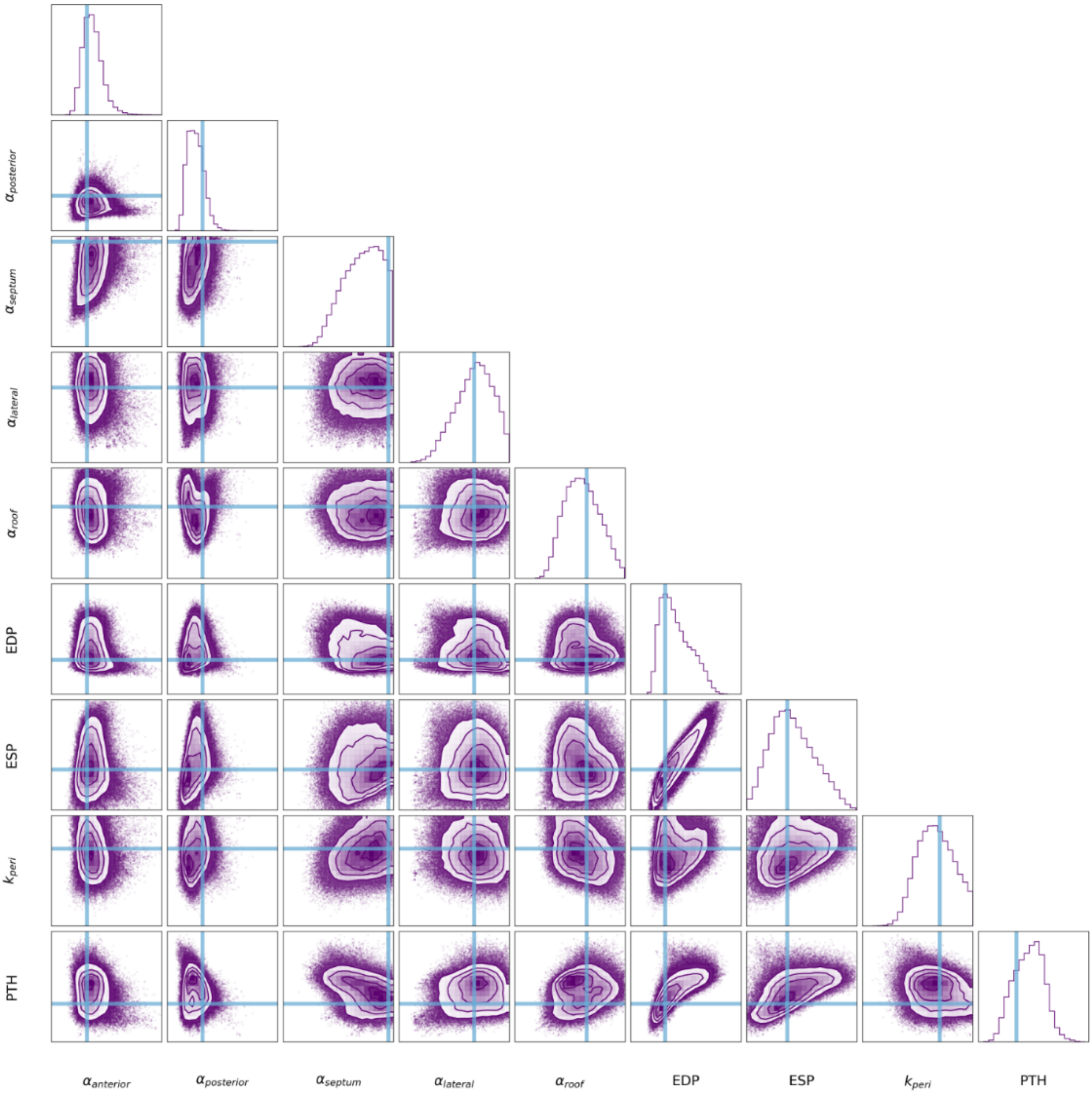

Fig 12: **Calibration using MCMC for case 02**. Plots showing the parameter distributions for case 02 estimated using MCMC. Each tile represents a projection of the Markov Chain samples. We implemented an ensemble sampler of 18 parallel walkers of 100 000 steps. A burn-in period of 10 000 steps and a thinning frequency of 10 was used. For each parameter, the MAP estimate is indicated by the blue line. The histograms along the diagonal display the posterior distributions for each parameter individually. The off-diagonal panels show the 2D distributions as contour plots where the contours are drawn at levels containing 11.8%, 39.3%, 67.5% and 86.4% of the samples.

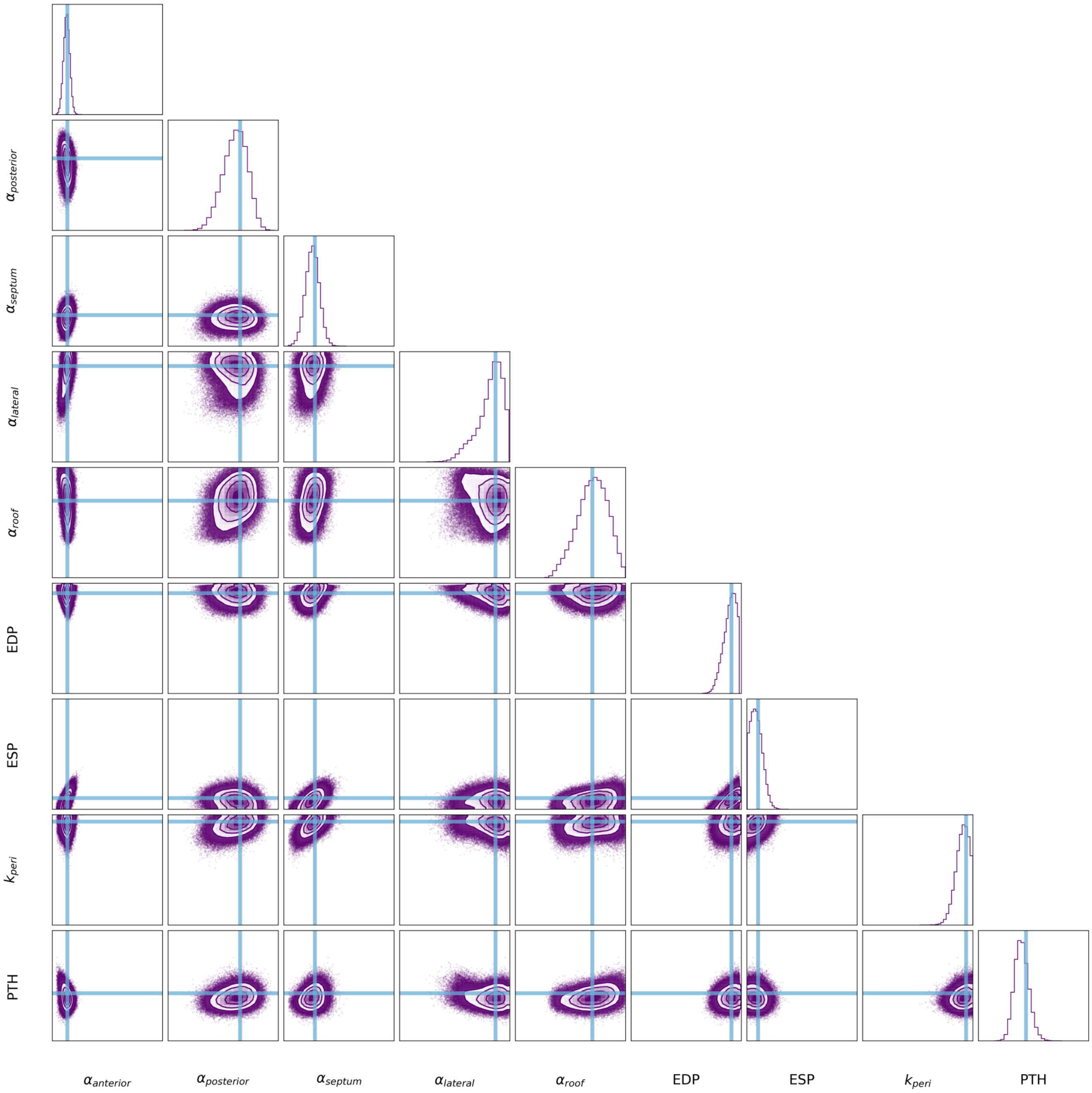

Fig 13: **Calibration using MCMC for case 03**. Plots showing the parameter distributions for case 03 estimated using MCMC. Each tile represents a projection of the Markov Chain samples. We implemented an ensemble sampler of 18 parallel walkers of 100 000 steps. A burn-in period of 10 000 steps and a thinning frequency of 10 was used. For each parameter, the MAP estimate is indicated by the blue line. The histograms along the diagonal display the posterior distributions for each parameter individually. The off-diagonal panels show the 2D distributions as contour plots where the contours are drawn at levels containing 11.8%, 39.3%, 67.5% and 86.4% of the samples.

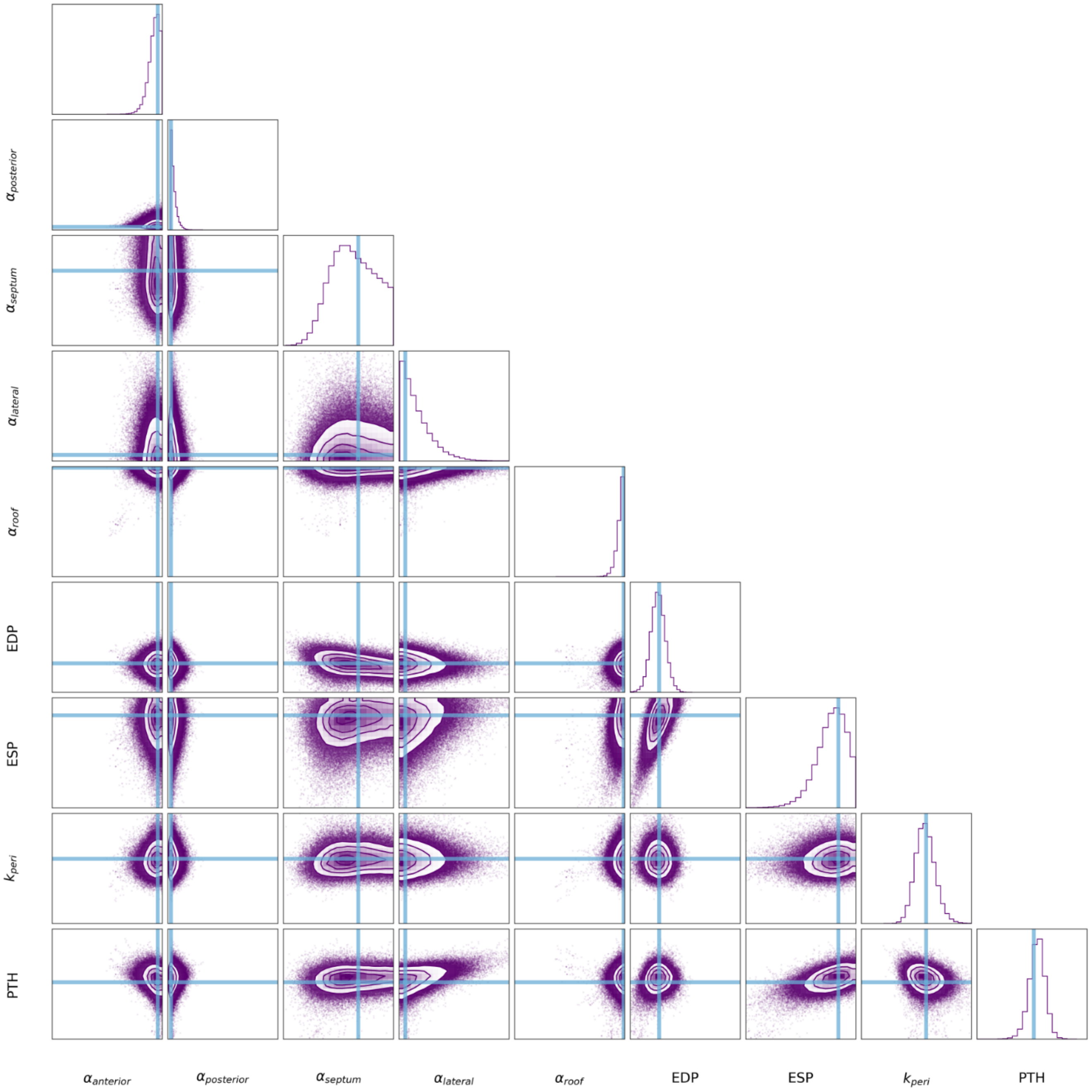

Fig 14: **Calibration using MCMC for case 04**. Plots showing the parameter distributions for case 04 estimated using MCMC. Each tile represents a projection of the Markov Chain samples. We implemented an ensemble sampler of 18 parallel walkers of 100 000 steps. A burn-in period of 10 000 steps and a thinning frequency of 10 was used. For each parameter, the MAP estimate is indicated by the blue line. The histograms along the diagonal display the posterior distributions for each parameter individually. The off-diagonal panels show the 2D distributions as contour plots where the contours are drawn at levels containing 11.8%, 39.3%, 67.5% and 86.4% of the samples.

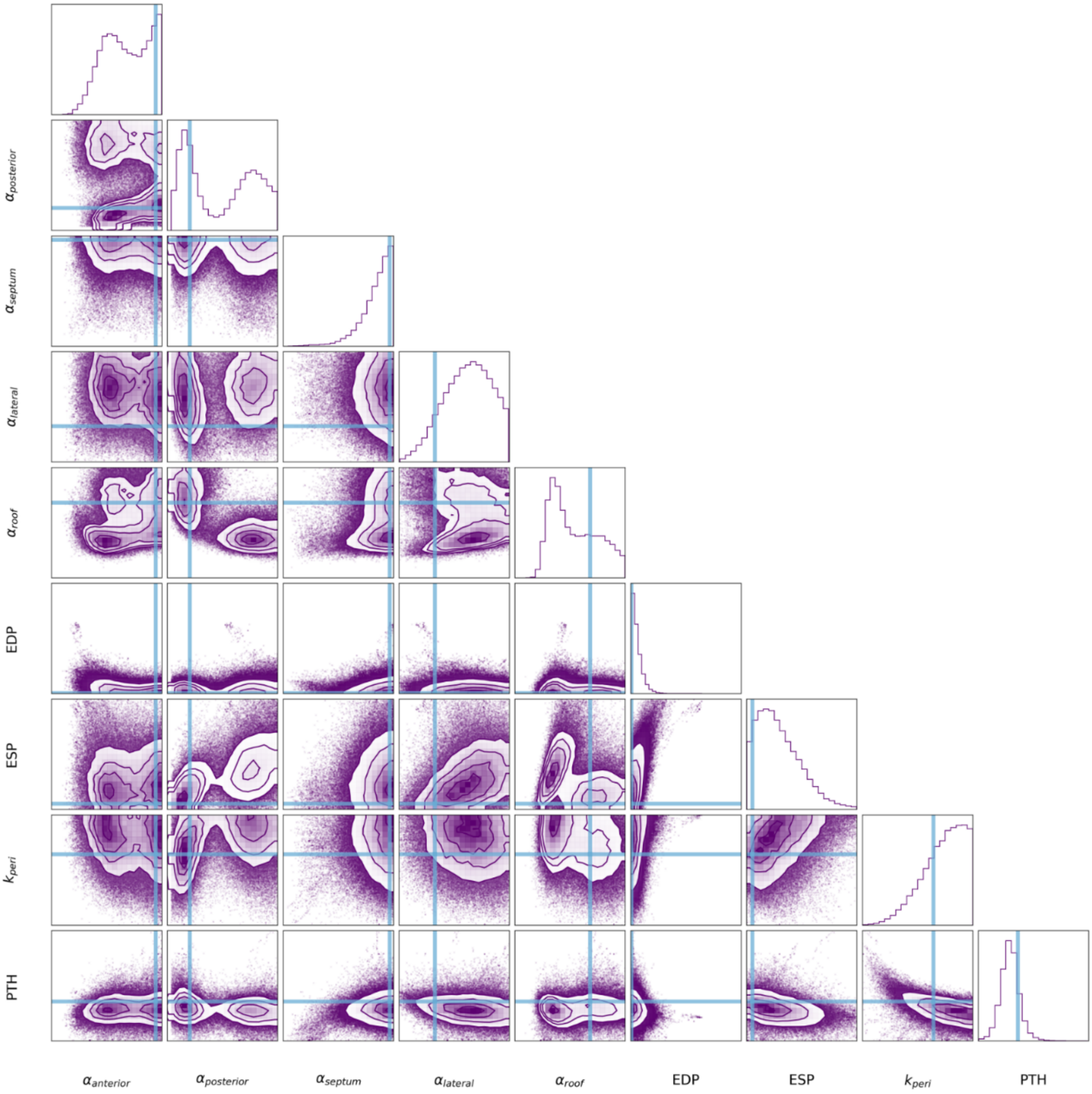

Fig 15: **Calibration using MCMC for case 05**. Plots showing the parameter distributions for case 05 estimated using MCMC. Each tile represents a projection of the Markov Chain samples. We implemented an ensemble sampler of 18 parallel walkers of 1 000 000 steps. A burn-in period of 910 000 steps and a thinning frequency of 10 was used. For each parameter, the MAP estimate is indicated by the blue line. The histograms along the diagonal display the posterior distributions for each parameter individually. The off-diagonal panels show the 2D distributions as contour plots where the contours are drawn at levels containing 11.8%, 39.3%, 67.5% and 86.4% of the samples.

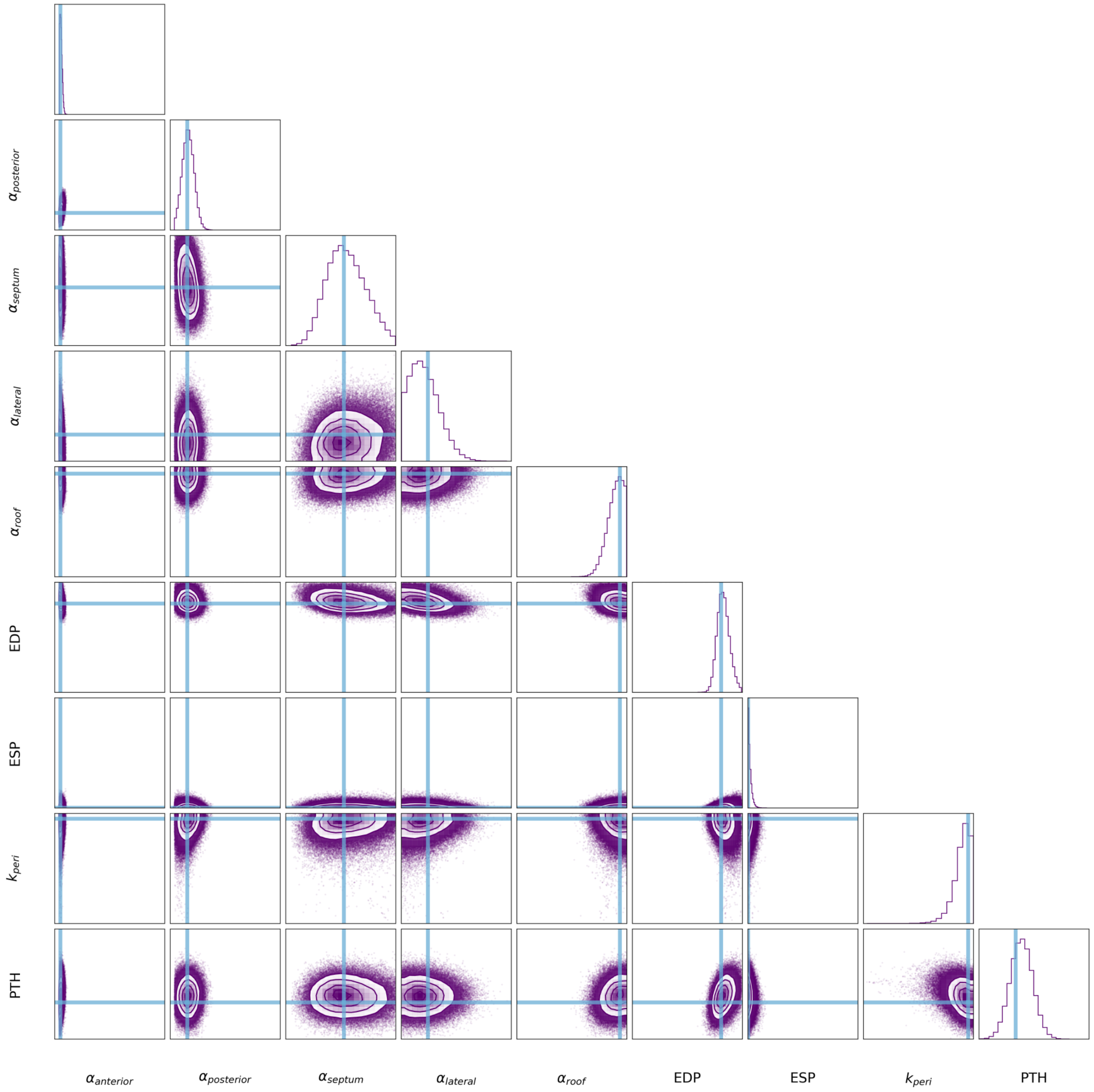

Fig 16: **Calibration using MCMC for case 06**. Plots showing the parameter distributions for case 06 estimated using MCMC. Each tile represents a projection of the Markov Chain samples. We implemented an ensemble sampler of 18 parallel walkers of 300 000 steps. A burn-in period of 210 000 steps and a thinning frequency of 10 was used. For each parameter, the MAP estimate is indicated by the blue line. The histograms along the diagonal display the posterior distributions for each parameter individually. The off-diagonal panels show the 2D distributions as contour plots where the contours are drawn at levels containing 11.8%, 39.3%, 67.5% and 86.4% of the samples.

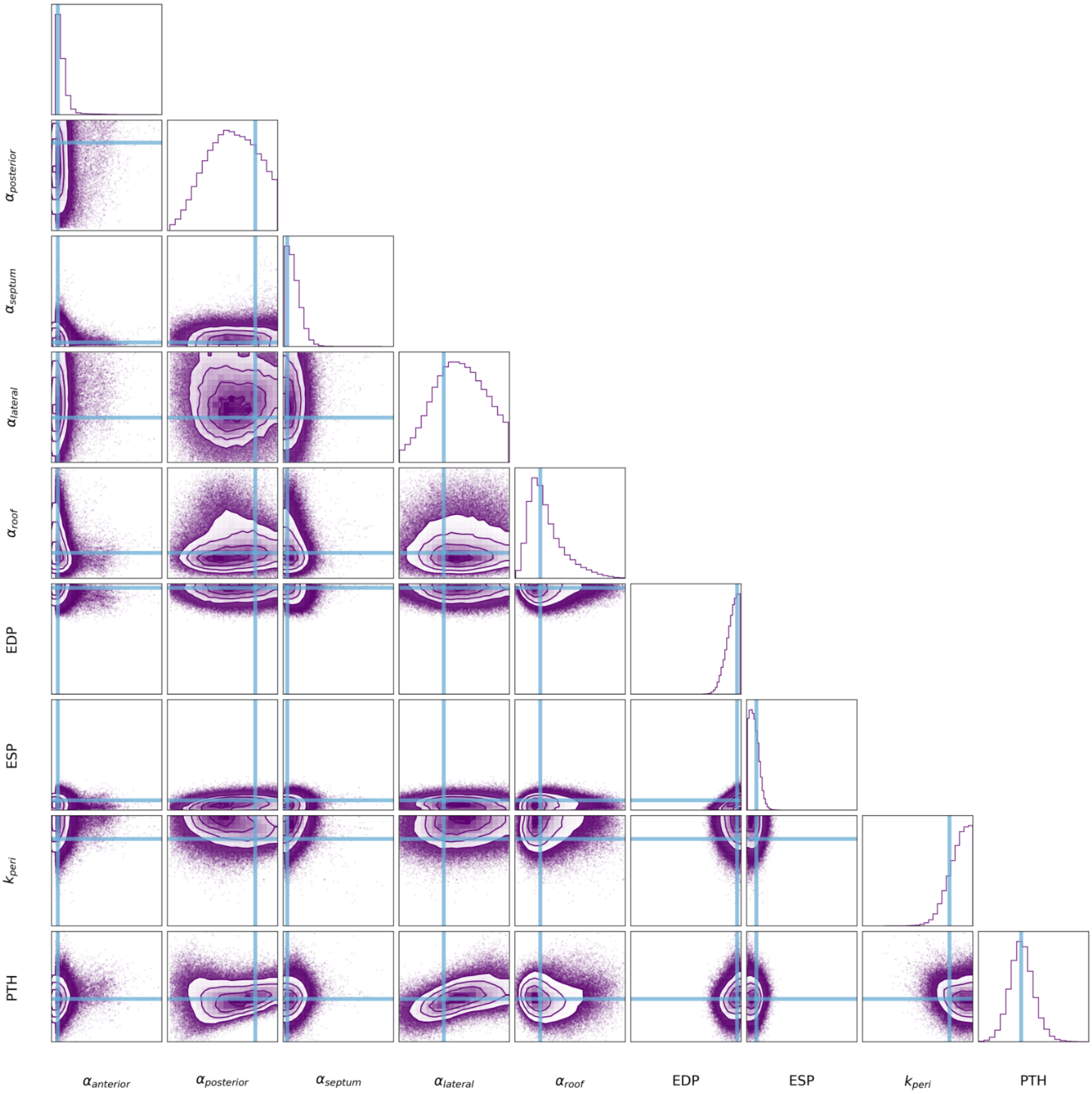

Fig 17: **Calibration using MCMC for case 07**. Plots showing the parameter distributions for case 07 estimated using MCMC. Each tile represents a projection of the Markov Chain samples. We implemented an ensemble sampler of 18 parallel walkers of 200 000 steps. A burn-in period of 110 000 steps and a thinning frequency of 10 was used. For each parameter, the MAP estimate is indicated by the blue line. The histograms along the diagonal display the posterior distributions for each parameter individually. The off-diagonal panels show the 2D distributions as contour plots where the contours are drawn at levels containing 11.8%, 39.3%, 67.5% and 86.4% of the samples.

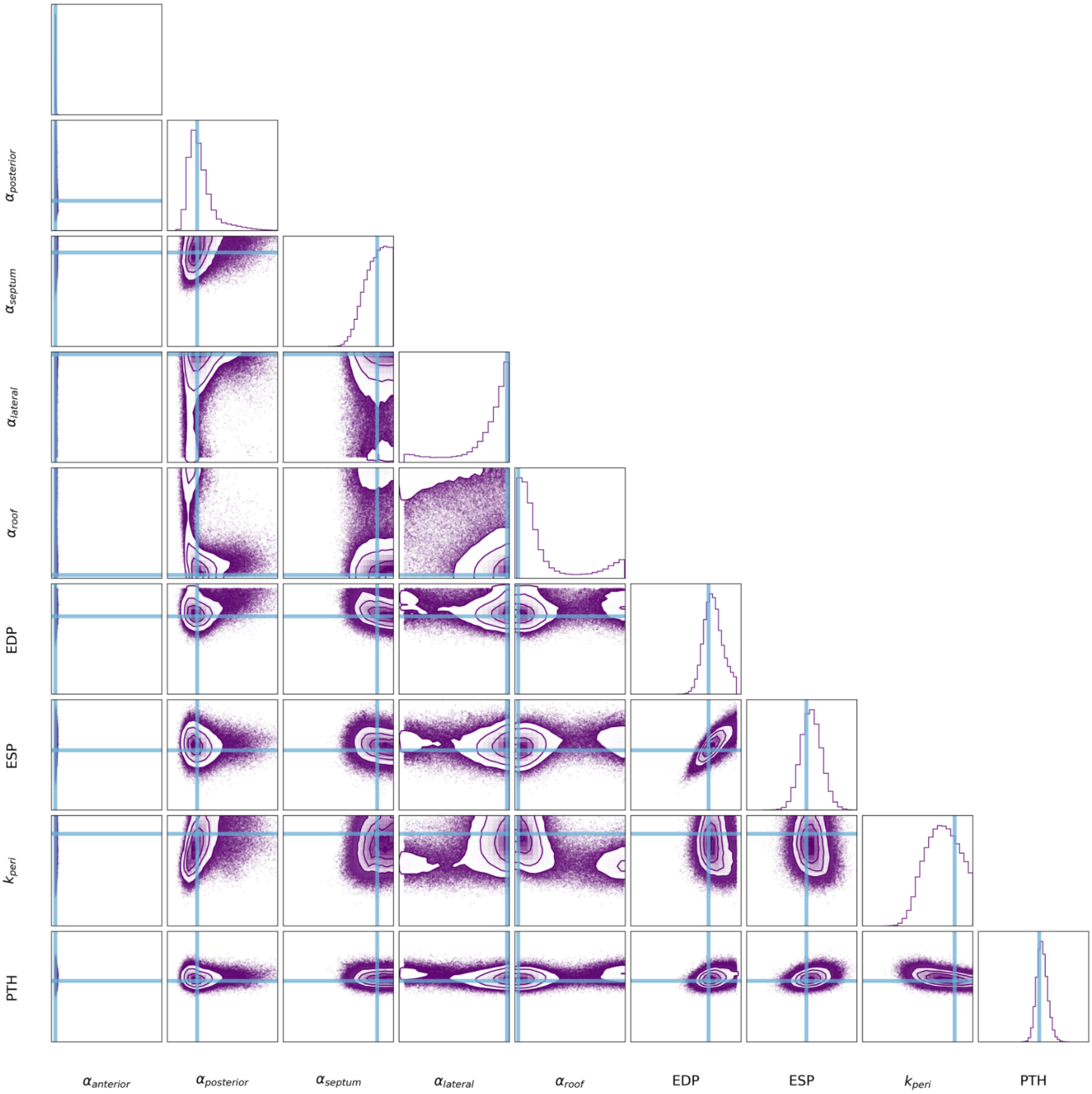

Fig 18: **Calibration using MCMC for case 08**. Plots showing the parameter distributions for case 08 estimated using MCMC. Each tile represents a projection of the Markov Chain samples. We implemented an ensemble sampler of 18 parallel walkers of 100 000 steps. A burn-in period of 10 000 steps and a thinning frequency of 10 was used. For each parameter, the MAP estimate is indicated by the blue line. The histograms along the diagonal display the posterior distributions for each parameter individually. The off-diagonal panels show the 2D distributions as contour plots where the contours are drawn at levels containing 11.8%, 39.3%, 67.5% and 86.4% of the samples.

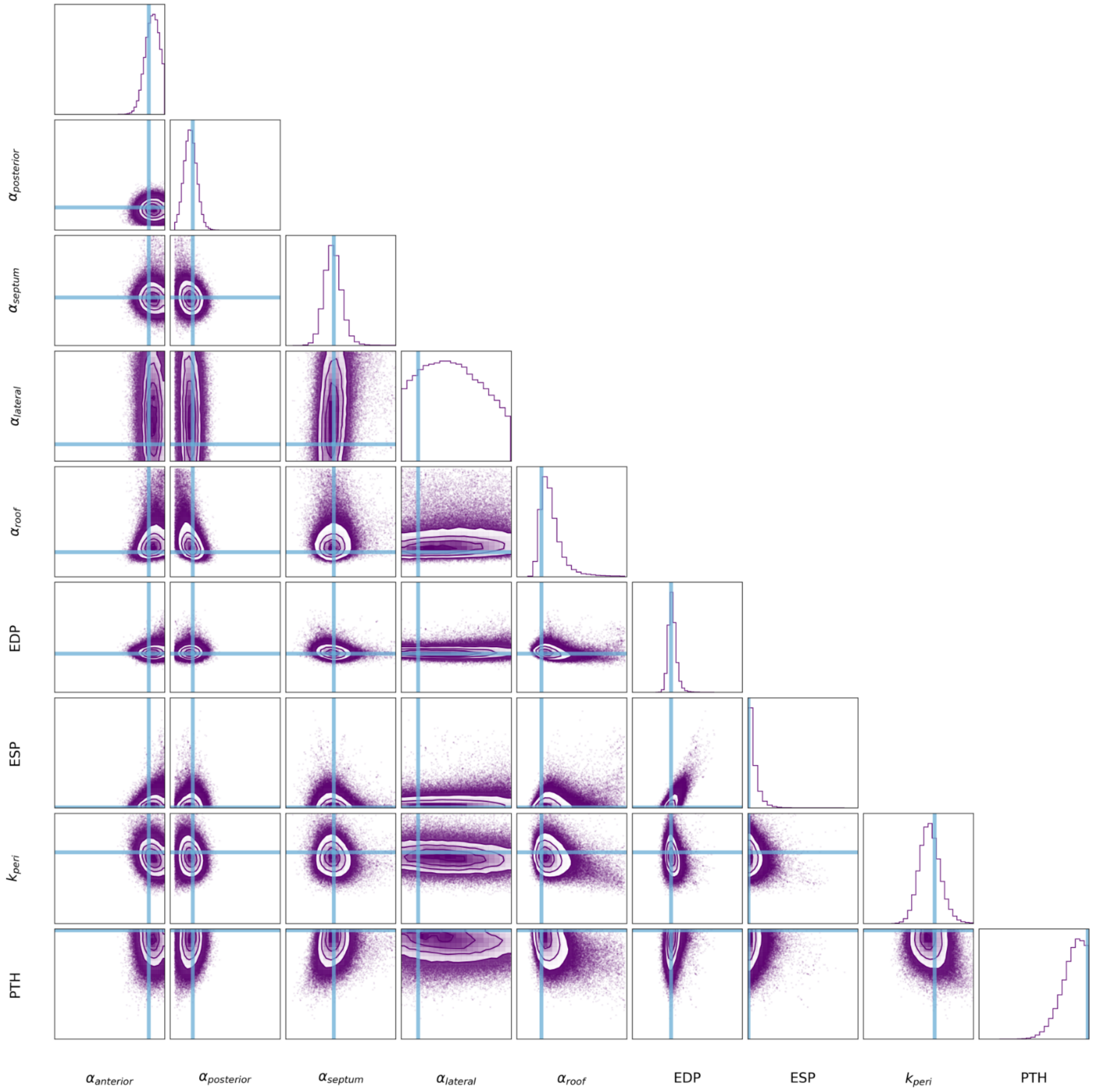

Fig 19: **Calibration using MCMC for case 09**. Plots showing the parameter distributions for case 09 estimated using MCMC. Each tile represents a projection of the Markov Chain samples. We implemented an ensemble sampler of 18 parallel walkers of 100 000 steps. A burn-in period of 10 000 steps and a thinning frequency of 10 was used. For each parameter, the MAP estimate is indicated by the blue line. The histograms along the diagonal display the posterior distributions for each parameter individually. The off-diagonal panels show the 2D distributions as contour plots where the contours are drawn at levels containing 11.8%, 39.3%, 67.5% and 86.4% of the samples.

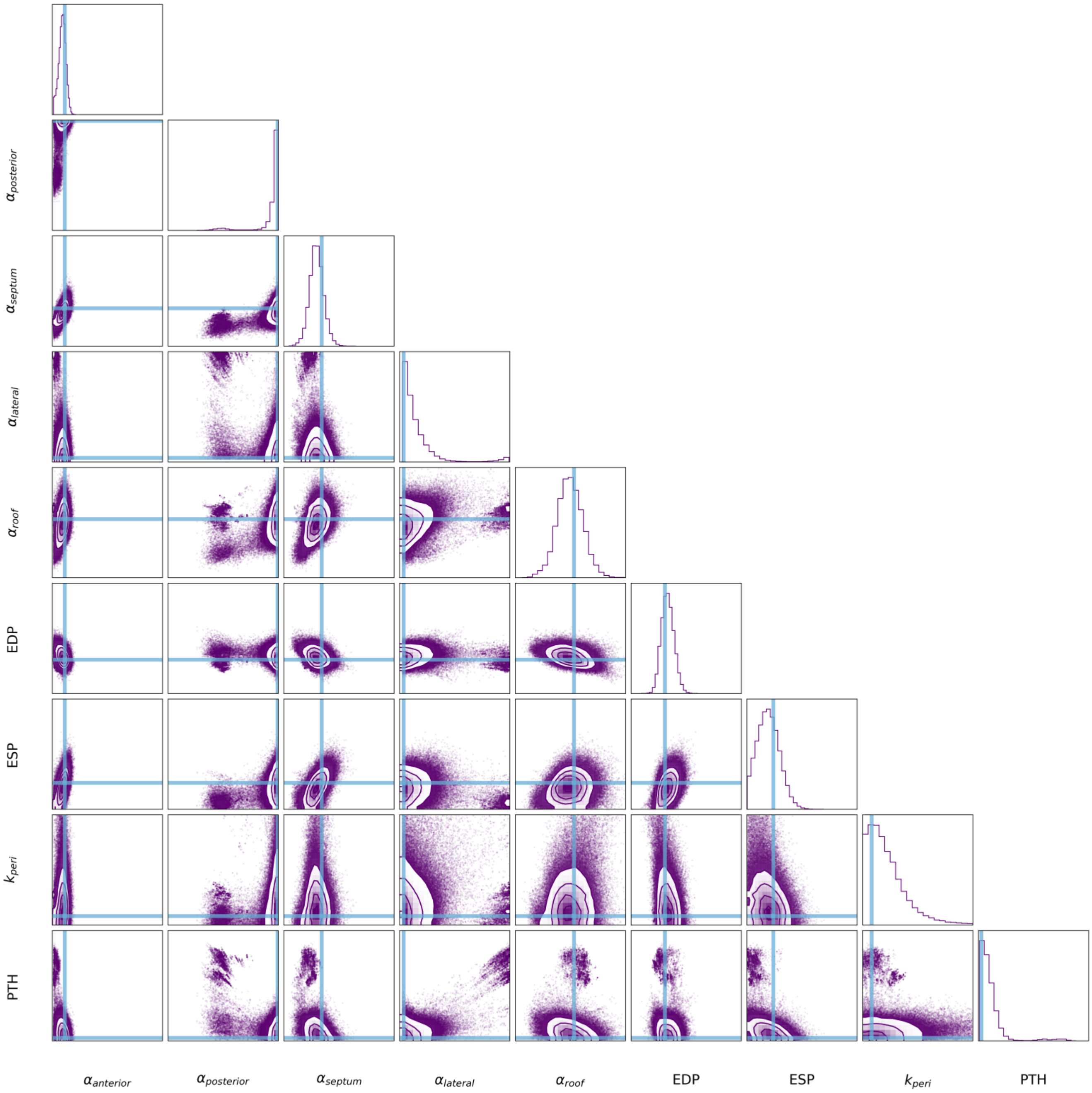

Fig 20: **Calibration using MCMC for case 10**. Plots showing the parameter distributions for case 10 estimated using MCMC. Each tile represents a projection of the Markov Chain samples. We implemented an ensemble sampler of 18 parallel walkers of 1 000 000 steps. A burn-in period of 910 000 steps and a thinning frequency of 10 was used. For each parameter, the MAP estimate is indicated by the blue line. The histograms along the diagonal display the posterior distributions for each parameter individually. The off-diagonal panels show the 2D distributions as contour plots where the contours are drawn at levels containing 11.8%, 39.3%, 67.5% and 86.4% of the samples.

To gain a better understanding of the performance of our calibration procedure, we examined posterior contraction. In the context of our study, posterior contraction refers to how the posterior distribution becomes increasingly concentrated around the patient-specific image-derived features, as more data are observed. Here, we compare the distribution arising from samples emulated from the MCMC-estimated posterior distribution to samples emulated from the first wave of HM. In Figs 21 to 30, we can observe how samples emulated from the posterior distribution form tight distributions that centered or closely centered around the image-derived values of the output features, while samples emulated from the initial uninformed parameter space exhibit wider distributions. Thus, from this framework, we obtained posterior distributions across all input parameters that recapitulate the desired clinical data. From this, we gained information about the relative differences in stiffness across regions, informing LA stiffness patterns.

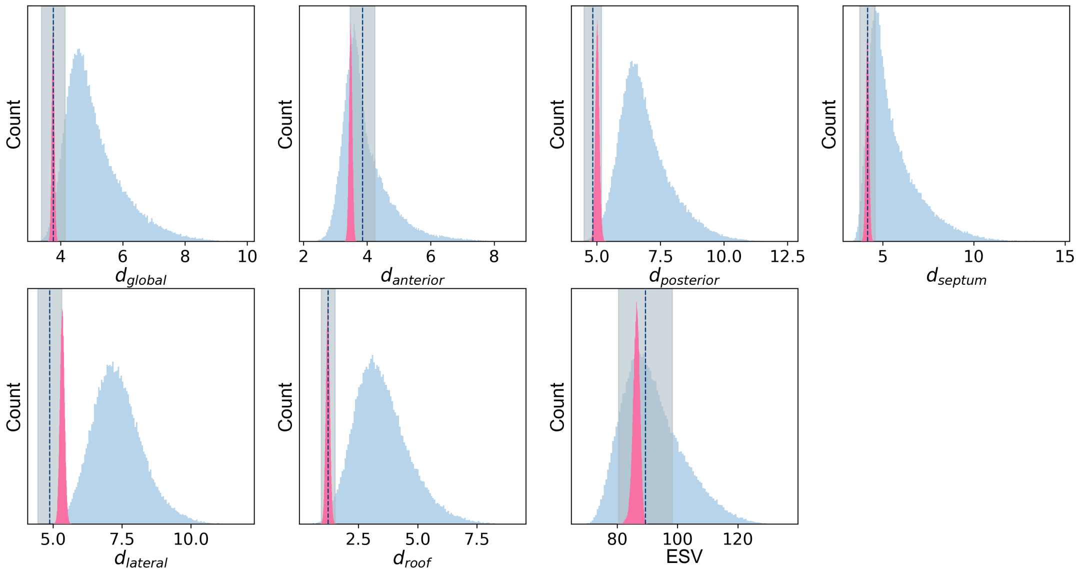

Fig 21: **Emulated samples for case 01**. The plots show how the emulated samples resulting from the posterior distribution obtained through MCMC (pink histograms) move towards the the image-derived output values (dashed line). The distribution of emulated samples coming from the first wave of HM are shown in light blue. The grey shaded areas represent ±2 SD of the image-derived target output values.

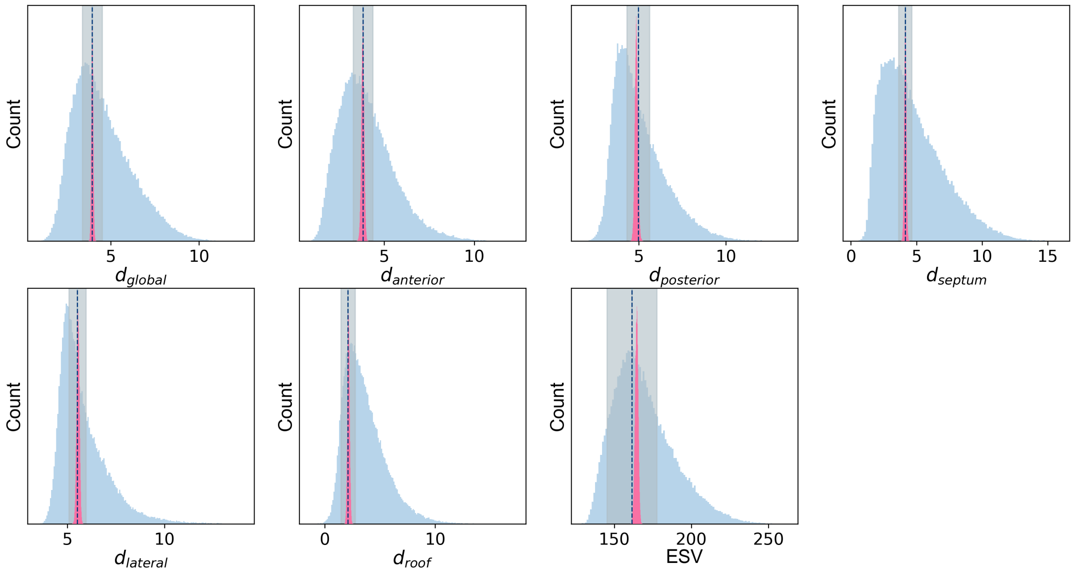

Fig 22: **Emulated samples for case 02**. The plots show how the emulated samples resulting from the posterior distribution obtained through MCMC (pink histograms) move towards the the image-derived output values (dashed line). The distribution of emulated samples coming from the first wave of HM are shown in light blue. The grey shaded areas represent ±2 SD of the image-derived target output values.

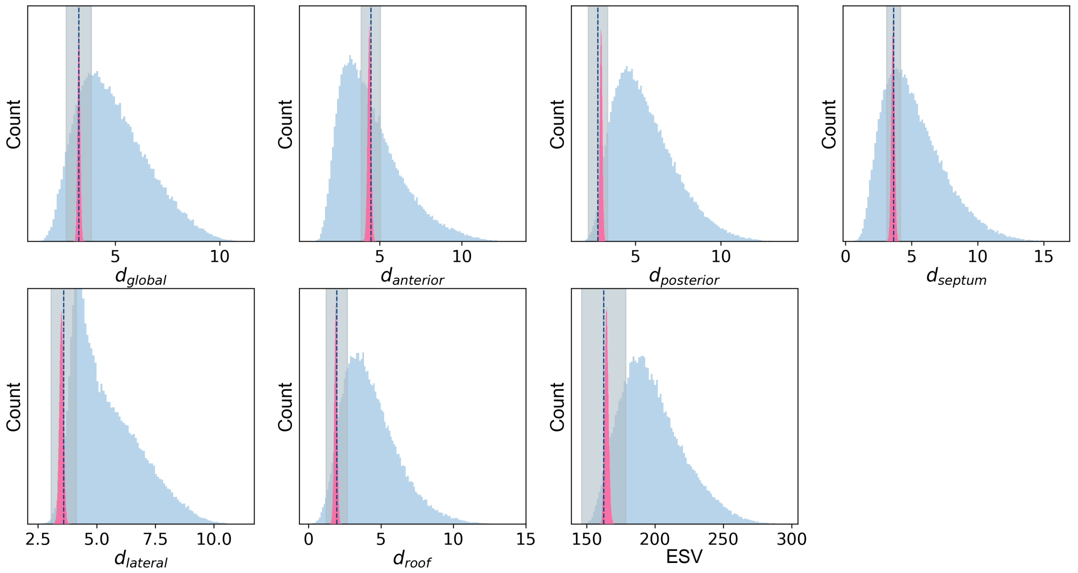

Fig 23: **Emulated samples for case 03**. The plots show how the emulated samples resulting from the posterior distribution obtained through MCMC (pink histograms) move towards the the image-derived output values (dashed line). The distribution of emulated samples coming from the first wave of HM are shown in light blue. The grey shaded areas represent ±2 SD of the image-derived target output values.

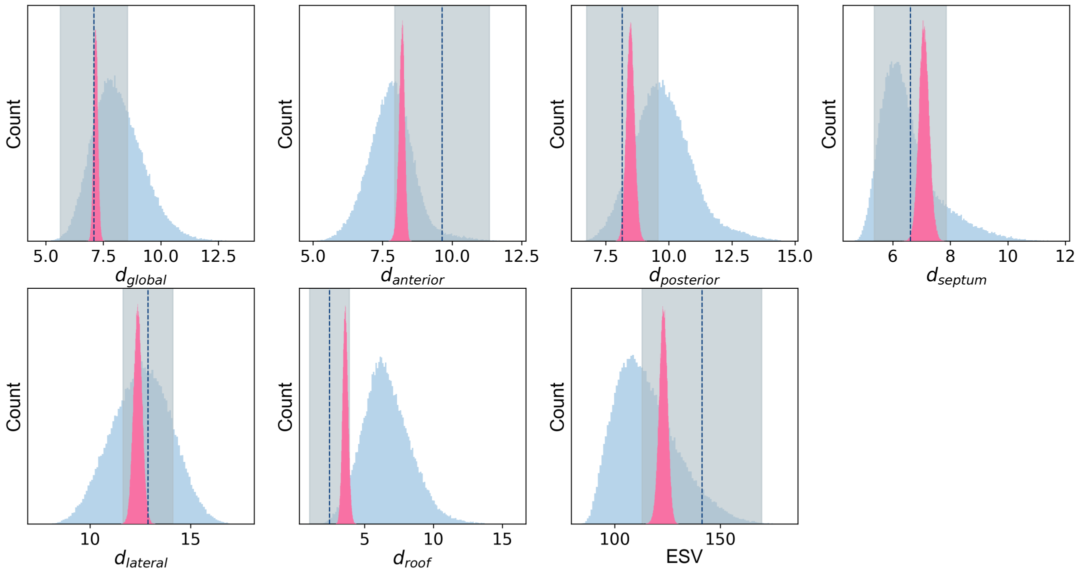

Fig 24: **Emulated samples for case 04**. The plots show how the emulated samples resulting from the posterior distribution obtained through MCMC (pink histograms) move towards the the image-derived output values (dashed line). The distribution of emulated samples coming from the first wave of HM are shown in light blue. The grey shaded areas represent ±2 SD of the image-derived target output values.

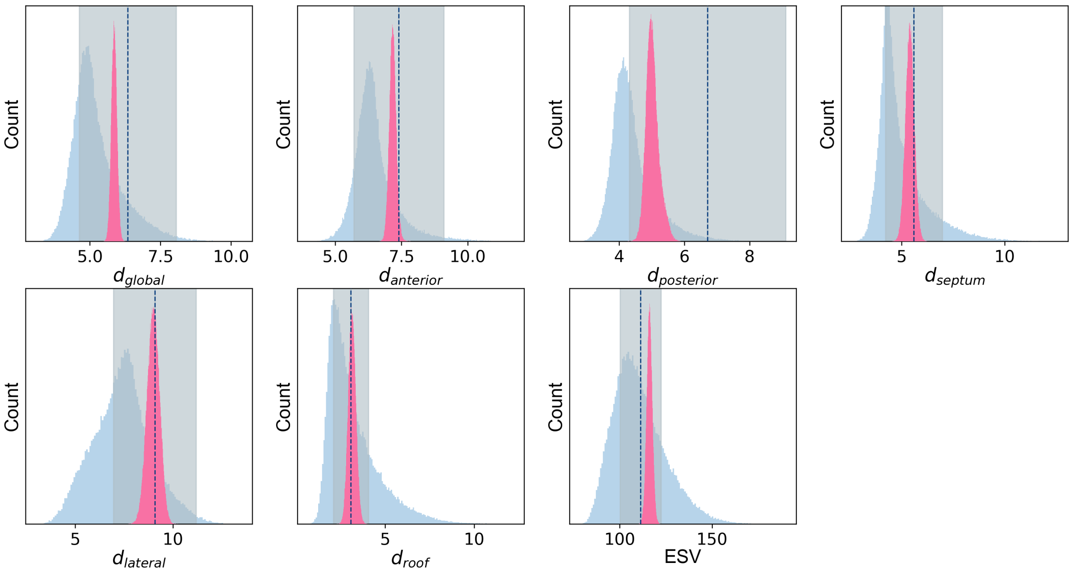

Fig 25: **Emulated samples for case 05**. The plots show how the emulated samples resulting from the posterior distribution obtained through MCMC (pink histograms) move towards the the image-derived output values (dashed line). The distribution of emulated samples coming from the first wave of HM are shown in light blue. The grey shaded areas represent ±2 SD of the image-derived target output values.

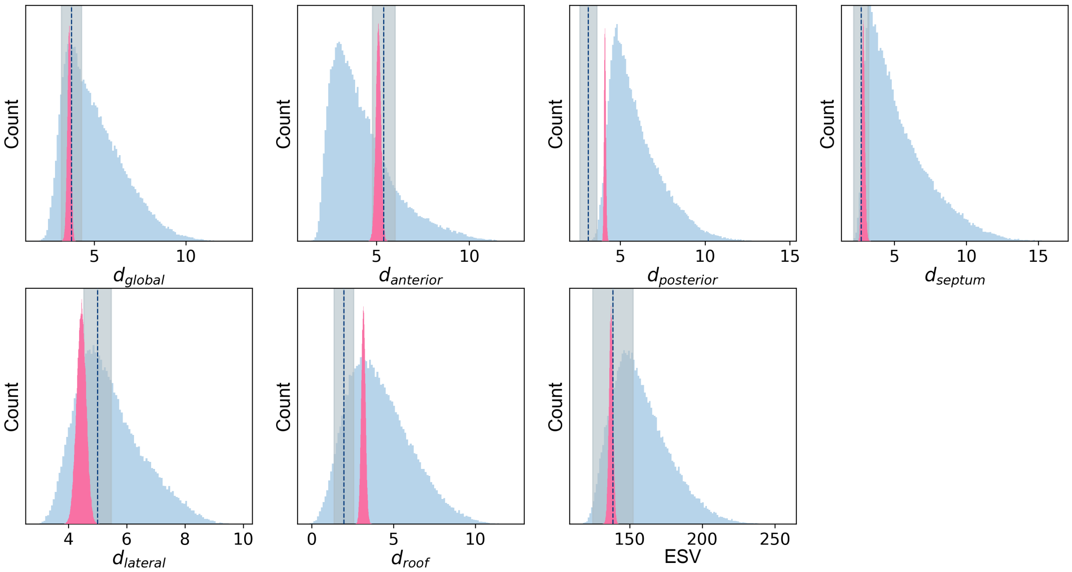

Fig 26: **Emulated samples for case 06**. The plots show how the emulated samples resulting from the posterior distribution obtained through MCMC (pink histograms) move towards the the image-derived output values (dashed line). The distribution of emulated samples coming from the first wave of HM are shown in light blue. The grey shaded areas represent ±2 SD of the image-derived target output values.

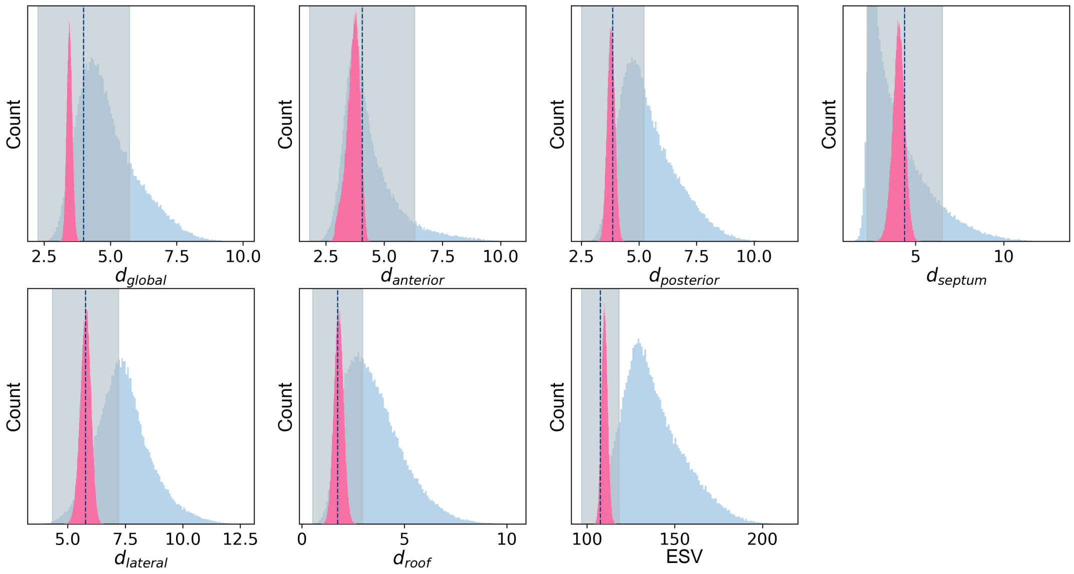

Fig 27: **Emulated samples for case 07**. The plots show how the emulated samples resulting from the posterior distribution obtained through MCMC (pink histograms) move towards the the image-derived output values (dashed line). The distribution of emulated samples coming from the first wave of HM are shown in light blue. The grey shaded areas represent ±2 SD of the image-derived target output values.

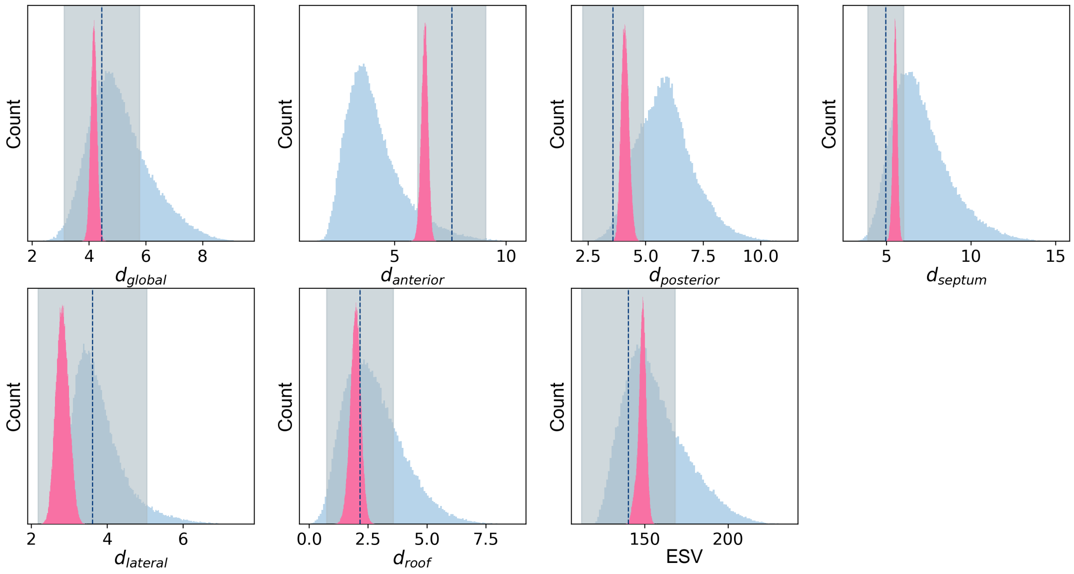

Fig 28: **Emulated samples for case 08**. The plots show how the emulated samples resulting from the posterior distribution obtained through MCMC (pink histograms) move towards the the image-derived output values (dashed line). The distribution of emulated samples coming from the first wave of HM are shown in light blue. The grey shaded areas represent ±2 SD of the image-derived target output values.

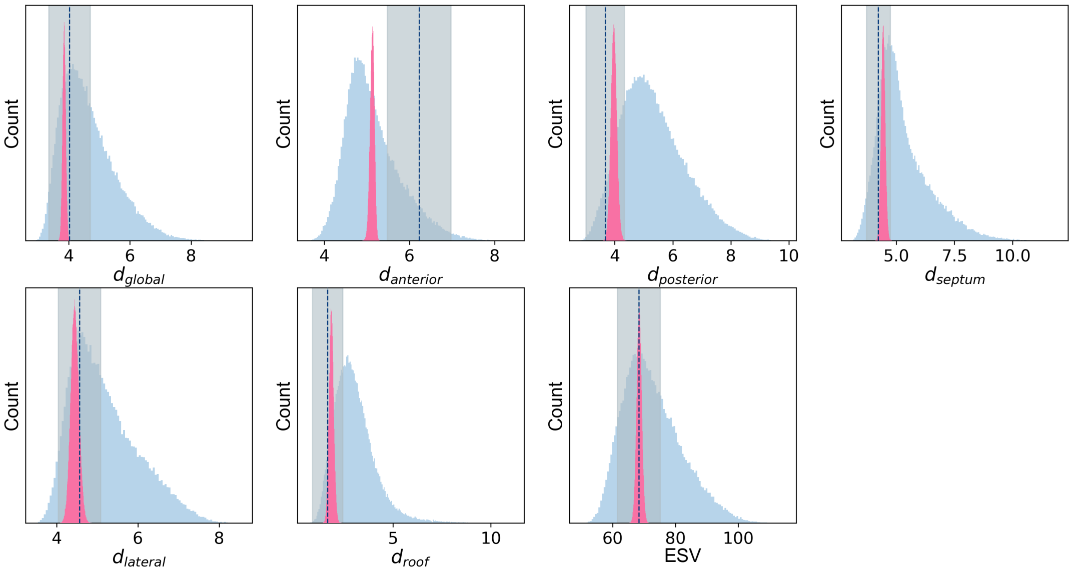

Fig 29: **Emulated samples for case 09**. The plots show how the emulated samples resulting from the posterior distribution obtained through MCMC (pink histograms) move towards the the image-derived output values (dashed line). The distribution of emulated samples coming from the first wave of HM are shown in light blue. The grey shaded areas represent ±2 SD of the image-derived target output values.

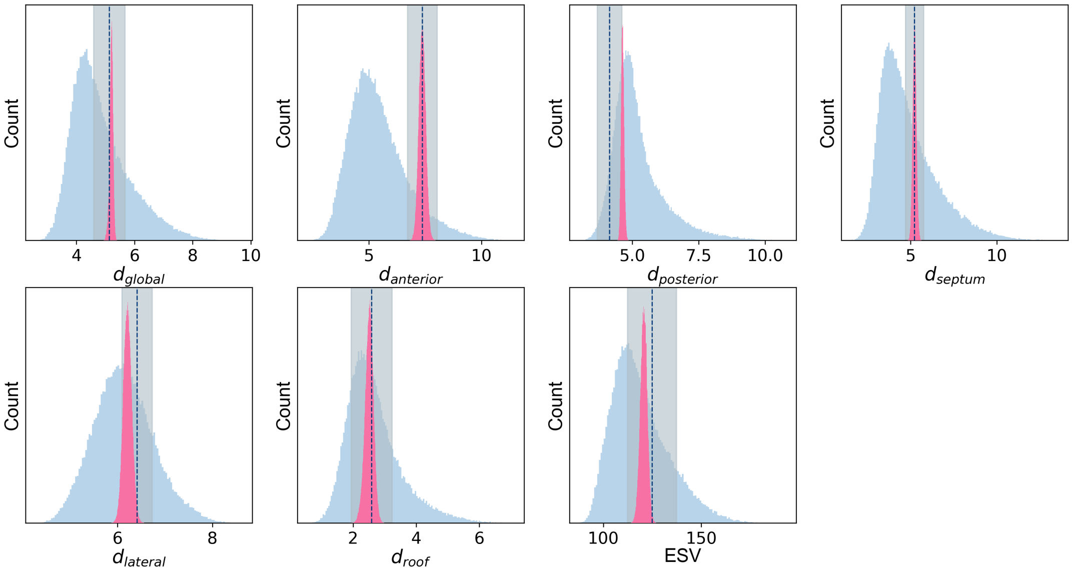

Fig 30: **Emulated samples for case 10**. The plots show how the emulated samples resulting from the posterior distribution obtained through MCMC (pink histograms) move towards the the image-derived output values (dashed line). The distribution of emulated samples coming from the first wave of HM are shown in light blue. The grey shaded areas represent ±2 SD of the image-derived target output values.

References

1. Rodero C, Strocchi M, Marciniak M, Longobardi S, Whitaker J, O'Neill MD, et al. Linking statistical shape models and simulated function in the healthy adult human heart. PLoS Computational Biology. 2021;17(4). doi:10.1371/journal.pcbi.1008851.
