# Supplementary material for: Regional heterogeneity in left atrial stiffness impacts passive deformation in a cohort of patient-specific models
Source: PLoS Comput Biol. 2025 Nov 5;21(11):e1013656. doi: 10.1371/journal.pcbi.1013656 (PMC12599961; doi:10.1371/journal.pcbi.1013656)
Supplement: S11 File — We calibrated our model to CT-derived deformations at ES. We validated the model against the global and regional deformation transients during the reservoir and conduit phases. (PDF) [file pcbi.1013656.s011.pdf]

Model validation

This study involved fitting model input parameters to ES displacements and volumes estimated from retrospective gated CT images. To validate our modelling framework, we compared the simulated global and regional displacement rates through the reservoir and conduit phases to that estimated from the CT motion models. This analysis ensured that physiological LA deformation was preserved throughout the simulated passive function and not only at the ES time-point.

Fig 1 highlights the phases of the cardiac cycle used for validation.

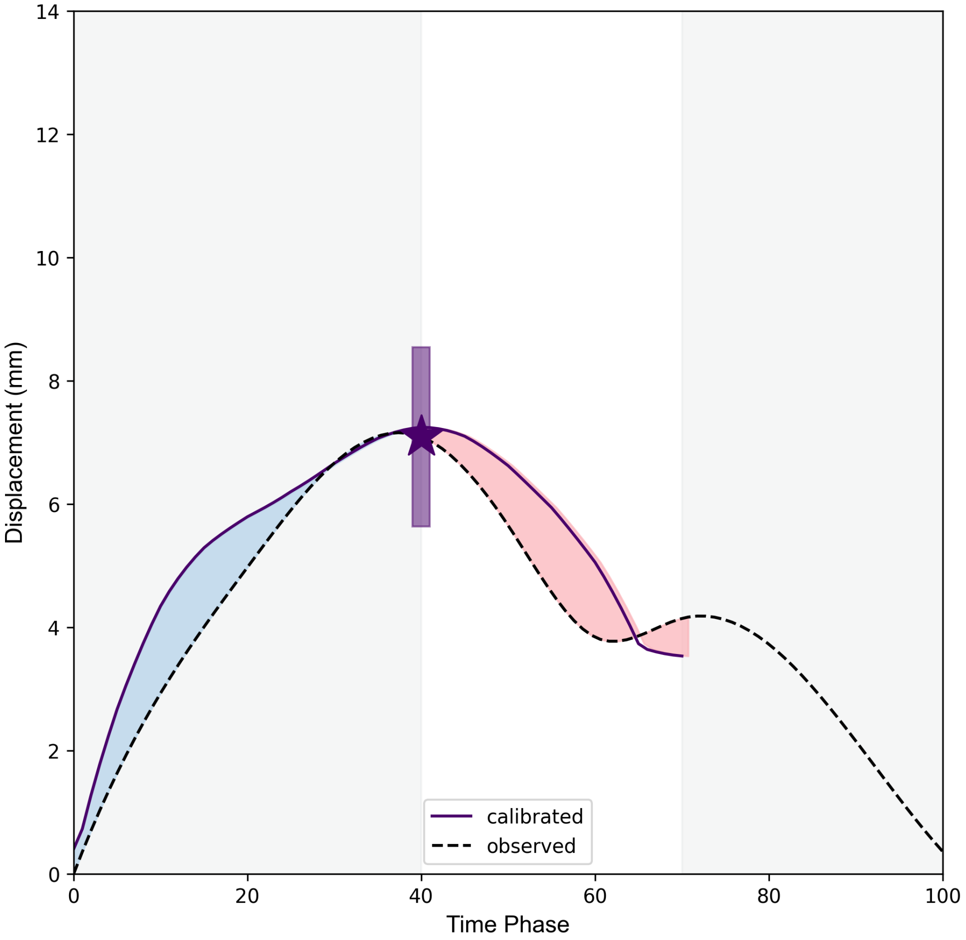

Fig 1: **Displacement rate assessment**. The phases of the cardiac cycle used for model validation. The reservoir phase is highlighted in blue while the conduit phase is highlighted in pink.

Fig 2 compares the simulated displacement rate values through the reservoir (panel A) and conduit (panel B) phases to those derived from the CT image set for each case. The average error in displacement rate over all regions through the reservoir phase was 0.48 ± 1.48 mm per unit time and through the conduit phase was -0.27 ± 0.57 mm per unit time.

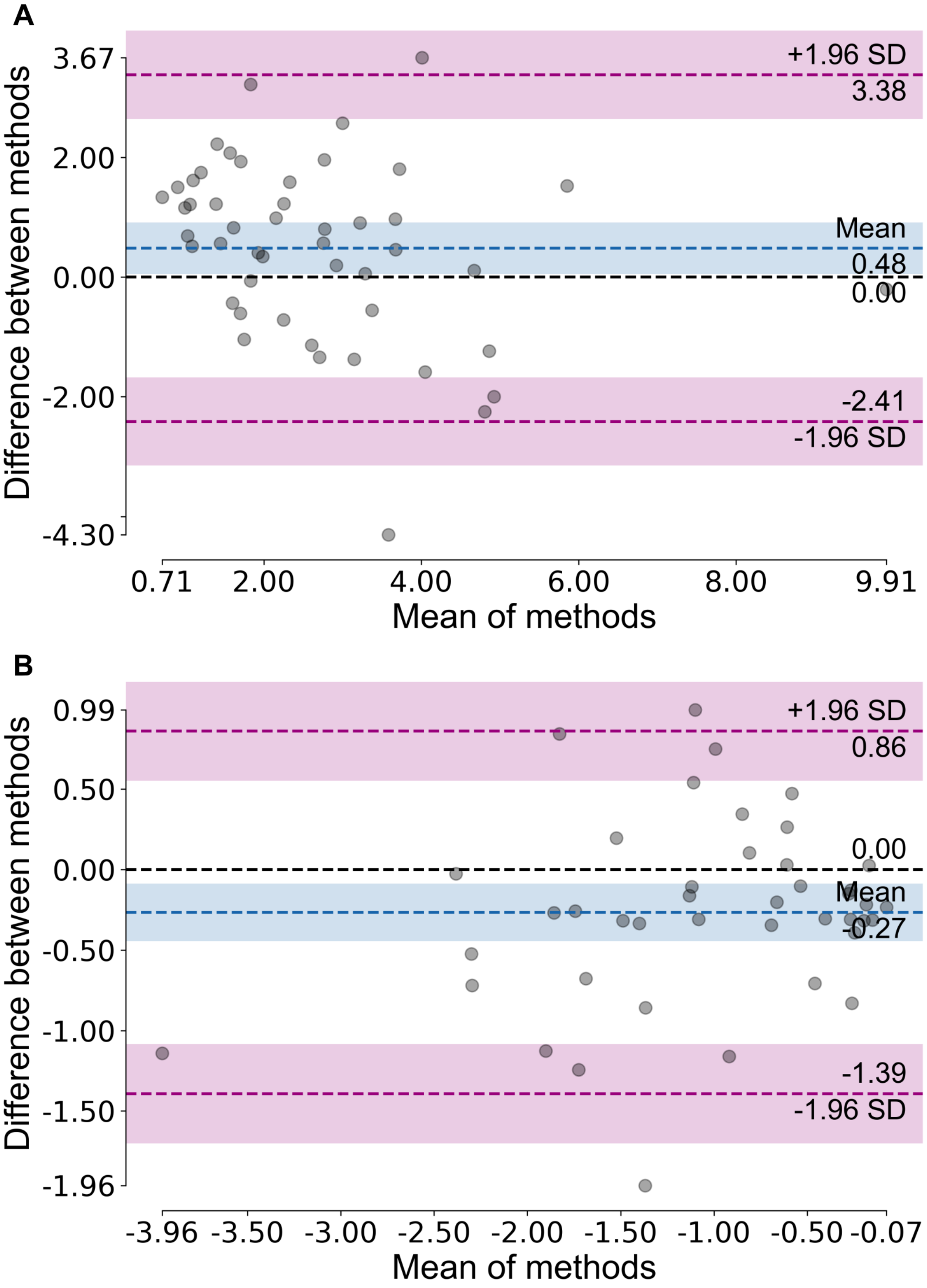

Fig 2: **Simulated vs CT-derived global displacement rate**. Using the final calibrated transients, the rate of change of displacement through the reservoir and conduit phases were compared to that of the CT-derived transients. The Bland-Altman plots show the difference in the global and regional displacement rates through the reservoir (A) and conduit (B) phase.
