# Supplementary material for: Regional heterogeneity in left atrial stiffness impacts passive deformation in a cohort of patient-specific models
Source: PLoS Comput Biol. 2025 Nov 5;21(11):e1013656. doi: 10.1371/journal.pcbi.1013656 (PMC12599961; doi:10.1371/journal.pcbi.1013656)
Supplement: S12 File — Comparison of the calibrated model performance to the baseline model when regional heterogeneity in myocardial stiffness is not considered and when a uniform LA wall thickness is assumed. (PDF) [file pcbi.1013656.s012.pdf]

# Calibration performance

In this study, our fitting procedure was carried out considering three different modelling scenarios:

1. Our baseline case that includes a patient-specific wall thickness and regional heterogeneity in myocardial stiffness.
2. A global stiffness case that includes patient-specific wall thickness and a single set of global stiffness parameters in each simulation.
3. A uniform thickness case that uses a uniform wall thickness of 2.0 mm. The wall thickness value chosen here is commonly chosen in biomechanical models [1].

Calibration accuracies was improved by the inclusion of regional heterogeneity in myocardial stiffness while the inclusion of patient-specific wall thickness, appeared less important to achieving the both the global and regional image-derived deformation.

Table 1 provides a summary of the discrepancy between the image-derived and simulated LA behaviour for each modelling scenario.

Table 1: The RMSE between the simulated and image-derived deformation from three model set-ups: (i) model includes patient-specific LA wall thickness and regionally varying stiffness parameters; (ii) model includes patient-specific LA wall thickness and a single global stiffness parameter; (iii) model includes uniform LA wall thickness and regionally varying stiffness parameters.

| | RMSE | | |
|---|---|---|---|
| | (i) | (ii) | (iii) |
| $d_{global}$ (mm) | 0.49254 | 0.852598 | 0.354982 |
| $d_{anterior}$ (mm) | 1.553045 | 1.777514 | 1.447884 |
| $d_{posterior}$ (mm) | 1.164333 | 1.825376 | 1.127184 |
| $d_{septum}$ (mm) | 0.863727 | 1.887971 | 0.991263 |
| $d_{lateral}$ (mm) | 0.732643 | 1.190151 | 0.922045 |
| $d_{roof}$ (mm) | 0.621185 | 1.088044 | 0.956135 |
| ESV (ml) | 11.03293 | 15.64566 | 11.26234 |

## References


1. Strocchi M, Gsell MAF, Augustin CM, Razeghi O, Roney CH, Prassl AJ, et al. Simulating ventricular systolic motion in a four-chamber heart model with spatially varying robin boundary conditions to model the effect of the pericardium. Journal of Biomechanics. 2020;101:109645. doi:10.1016/J.JBIOMECH.2020.109645.
