# Supplementary material for: Regional heterogeneity in left atrial stiffness impacts passive deformation in a cohort of patient-specific models
Source: PLoS Comput Biol. 2025 Nov 5;21(11):e1013656. doi: 10.1371/journal.pcbi.1013656 (PMC12599961; doi:10.1371/journal.pcbi.1013656)
Supplement: S13 File — We used a hemispherical geometry and simplified boundary conditions to examine the relationship between wall thickness and deformation. (PDF) [file pcbi.1013656.s013.pdf]

Association between wall thickness and deformation in simplified geometry

The complex geometry of the LA coupled with its physiological loading constraints likely have important consequences on observable LA biomechanics. Using a simplified geometry and loading constraints, we can gain insight into the role that these boundary conditions may play on the apparent relationships between anatomical features such as wall thickness, and biomechanics. We simulated the inflation of a hemisphere with varying wall thickness. In this model, the wall thickness randomly varied between 1 mm and 5 mm across the geometry. The hemisphere was closed using a flat plane and a ring was added to the hemisphere annulus. The simulation set-up and boundary conditions applied are shown in Fig 1. To hold the geometry in place during deformation, the hemispherical geometry was constrained using an omnidirectional spring of stiffness 0.001 kPa/μm applied to the annular region. We also applied normal springs on the outer surface of the geometry to mimic the effect of the pericardium, consistent with the boundary conditions applied to the patient-specific LA geometries. Similar to the main manuscript, the stiffness of the normal pericardial springs varied spatially over the geometry surface such that the greatest constraint of 0.0005 kPa/μm was applied to the geometry roof and the area around the base was free to move. The transversely isotropic Guccione material law was used to describe the passive mechanical behaviour of the hemisphere. The material parameter values were $C = 1.7$ kPa, b_f =8, $b_{ft} = 4$, b_t =3 as determined in Nasopoulou et al [1] as estimates for healthy LV myocardium. For simplicity, fibre and sheet directions were defined using a ventricular rule-based method, where ventricular endocardium and epicardium fibre directions were set to 60° and -60°, respectively. The endocardial sheet direction was set to -65° and the epicardial sheet direction was set to 25° [2]. For the remaining areas, which included the annular ring as well as plane closing the hemisphere geometry, the passive mechanics were represented by the non-linear isotropic Neo-Hookean constitutive law. For the annular ring, we used $c = 7.45$ kPa, analogous to the pulmonary vein rings in our LA simulations. In the plane, $c = 1000$ kPa to restrict its deformation during inflation. In all regions, incompressibility was enforced by assigning a bulk modulus of $\kappa = 1000$ kPa.

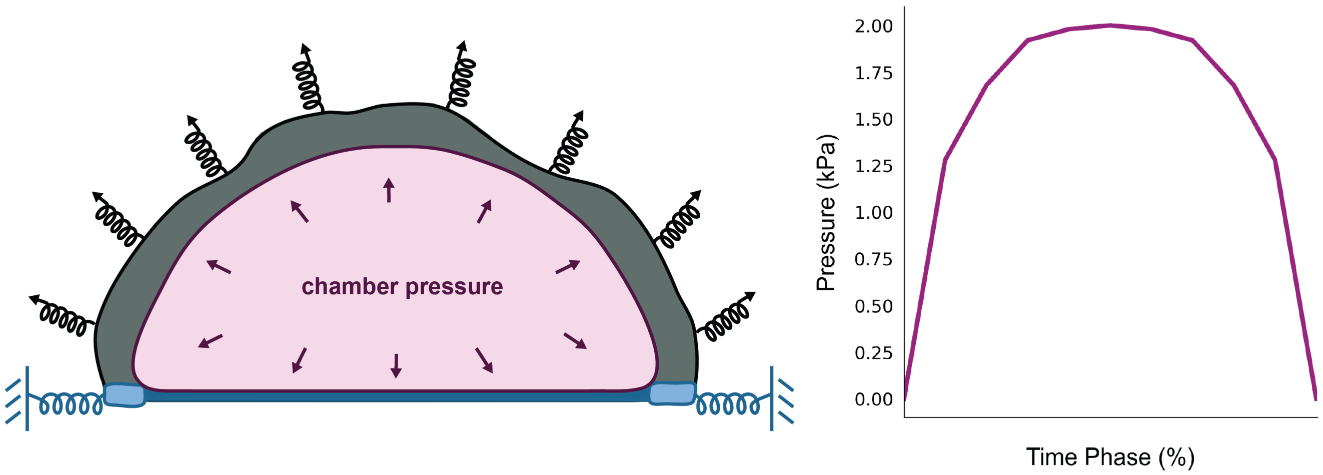

Fig 1: **Inflation model using a hemishpere with varying wall thickness**. The simulation set-up and loading conditions applied in our simplified modelling set-up.

The geometry was inflated to a peak pressure of 2 kPa. We used the displacement and principal strains measured at the point of peak inflation to investigate the relationship between wall thickness and deformation. Fig 2 illustrates our findings. The plot in Fig 2, panel A does not show a clear correlation between wall thickness and displacement nor between wall thickness and strain. When we remove the effect of the pericardium as shown in Fig 2, panel B, keeping all other simulation conditions constant, again, there appears to be no association between deformation, characterised by displacement or strain, and wall thickness.

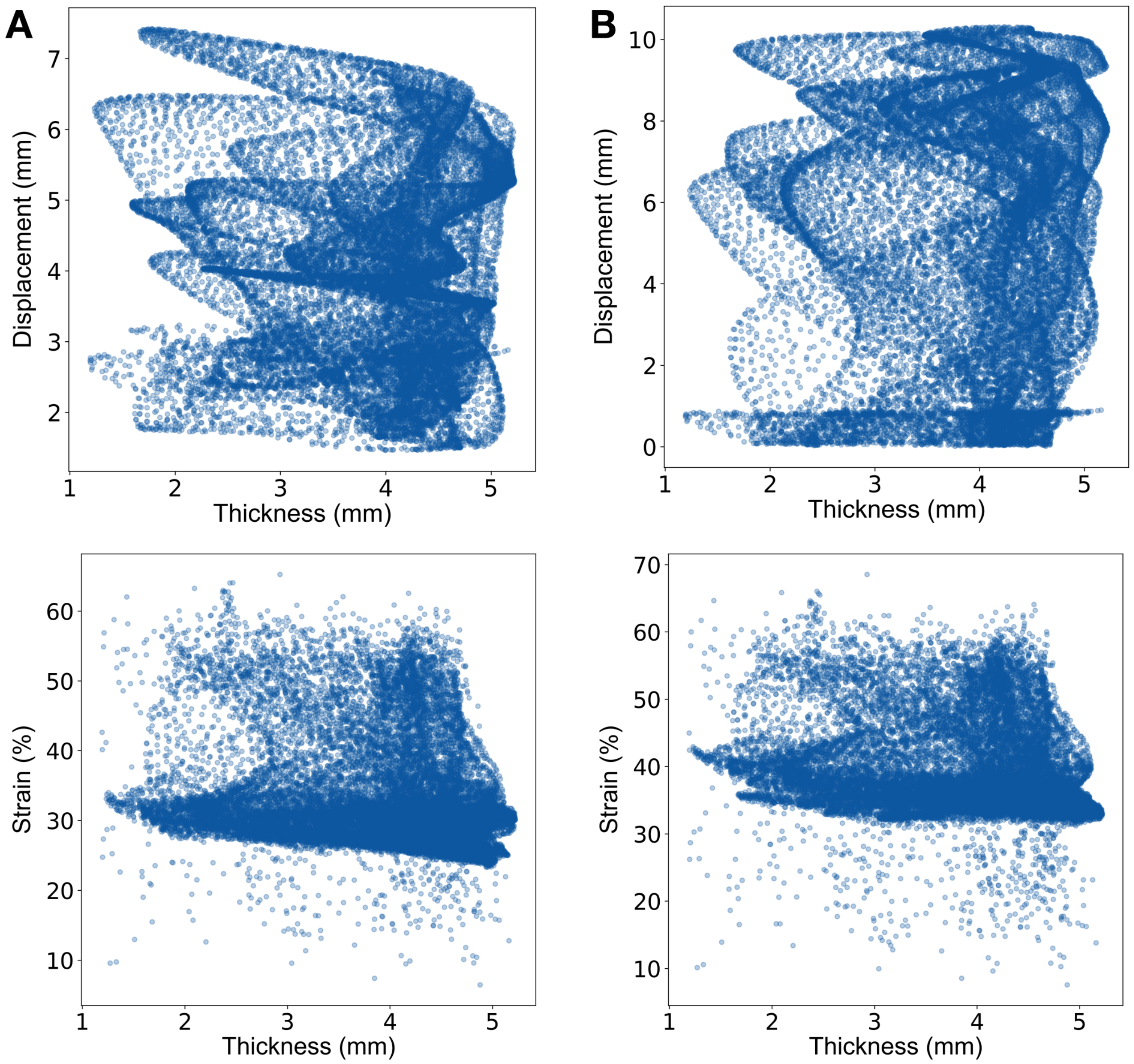

Fig 2: **Effect of wall thickness on simulated displacement**. Plots shows how displacement and strain at the point of peak inflation varies with wall thickness with (A) and without (B) the effect of a pericardium.

To investigate the potential link between wall thickness and deformation further, we divided the hemisphere into three regions and explored deformation an thickness regionally. Fig 4 shows our findings. After splitting the hemisphere into regions as shown in Fig 3 , we did not observe any correlation between deformation (displacement and strain) and wall thickness across the regions, with (Fig 4, panel A) or without (Fig 4, panel B) the pericardium being present.

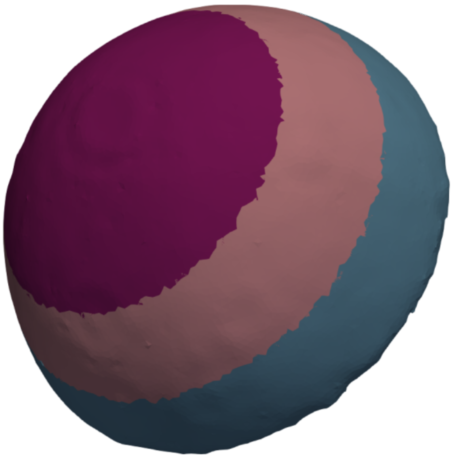

Fig 3: **Region definition in hemisphere model**. The hemispherical geometry was divided into 3 regions using a longitudinal Laplace-based coordinate system where 0 was defined at the geometry roof and 1 was defined as the area around the annular ring.

However, in Fig 4, panel B, we can see an increase in regional displacements moving further away from the simulation boundary constraint at the annular ring. Additionally, in Figs 2 and 4, the presence of the pericardium restricts the magnitude of deformation. These results suggest that the association between wall thickness and deformation may be highly dependent on anatomy, the location of thicker regions and physical constraints and boundary conditions. These confounding factors may weaken any apparent correlation between wall thickness and deformation.

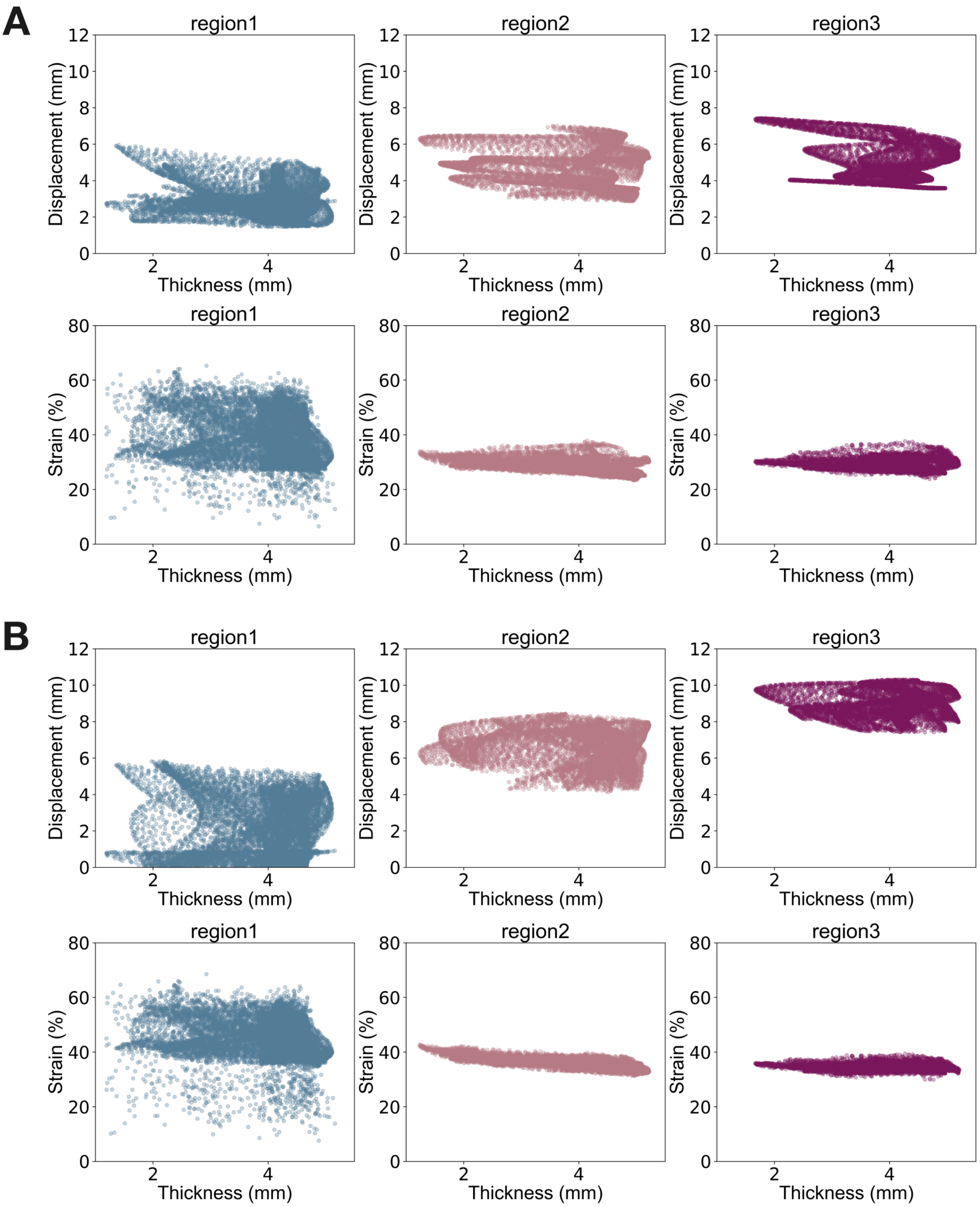

Fig 4: **Regional associations of wall thickness and simulated deformation**. The hemispherical geometry was divided into 3 regions. Plots shows how displacement and strain at the point of peak inflation varies with wall thickness across the defined regions with (A) and without (B) the effect of a pericardium.

References

1. Nasopoulou A, Shetty A, Lee J, Nordsletten D, Rinaldi CA, Lamata P, et al. Improved identifiability of myocardial material parameters by an energy-based cost function. Biomechanics and Modeling in Mechanobiology. 2017;16(3):971–988. doi:10.1007/S10237-016-0865-3.

2. Bayer JD, Blake RC, Plank G, Trayanova NA. A novel rule-based algorithm for assigning myocardial fiber orientation to computational heart models. Annals of Biomedical Engineering. 2012;40(10). doi:10.1007/s10439-012-0593-5.
