# Supplementary material for: Regional heterogeneity in left atrial stiffness impacts passive deformation in a cohort of patient-specific models
Source: PLoS Comput Biol. 2025 Nov 5;21(11):e1013656. doi: 10.1371/journal.pcbi.1013656 (PMC12599961; doi:10.1371/journal.pcbi.1013656)
Supplement: S14 File — We examined the suitability of the law of Laplace for application to the LA. (PDF) [file pcbi.1013656.s014.pdf]

Consideration of the Law of Laplace

In the law of Laplace, wall stress is proportional to the ratio of the radius of curvature and wall thickness.

The law of Laplace states that wall stress (σ) in a hollow thin-walled pressure chamber is directly proportional to pressure (P) and the radius of curvature (r), and inversely proportional to wall thickness (WT) [1]:

$$\sigma = \frac{P \cdot r}{2 \cdot WT}. \tag{1}$$

In the LA, the law of Laplace may provide a reasonable approximation for LA wall stress. Assuming a simple linear stiffness model, LA wall stress is proportional to LA wall strain:

$$\sigma = E \cdot \varepsilon. \tag{2}$$

Assuming a constant pressure and radius of curvature over the LA, we may expect to observe an inverse linear dependency of regional LA stiffness on the product of regional wall thickness and strain:

$$E_{region} \propto \frac{1}{\varepsilon_{region} \cdot WT}. \tag{3}$$

Using mixed effect models, we did not find that the inverse product of wall thickness and strain was a significant predictor of regional stiffness (p = 0.312). Fig 1 shows a scatterplot of this data.

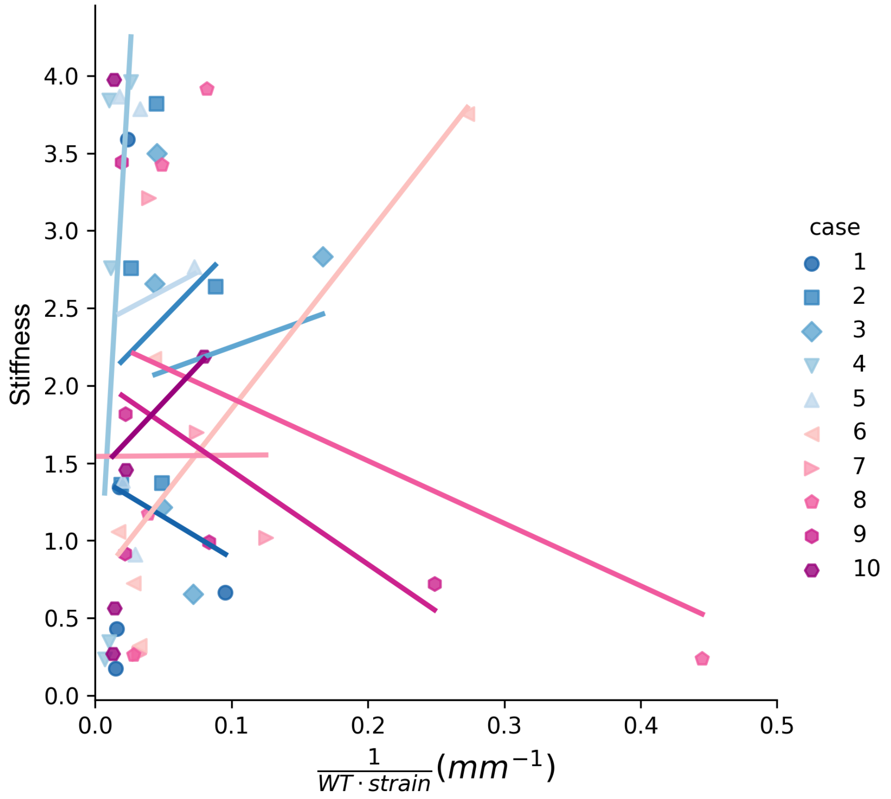

Fig 1: **Correlation using the law of Laplace**. Plots show how regional stiffness parameters vary with the inverse product of regionally averaged LA wall thickness and regional averages of end-systolic (ES) strain. Each marker symbol corresponds to one of the 10 patients.

This result suggests that the Laplace law is not a good model for the LA. This is likely due to the restrictive boundary conditions on the mitral valve, pulmonary veins and pericardium that constrain chamber deformation and are not accounted for in the law of Laplace.

References

1. Valentinuzzi ME, Kohen AJ. Laplaces law: What it is about, where it comes from, and how it is often applied in physiology [retrospectroscope]. IEEE Pulse. 2011;2(4). doi:10.1109/MPUL.2011.942054.
